# Melanosomal formation of PMEL core amyloid is driven by aromatic residues

**DOI:** 10.1038/srep44064

**Published:** 2017-03-08

**Authors:** Jia Shee Hee, Susan M. Mitchell, Xinran Liu, Ralf M. Leonhardt

**Affiliations:** 1 Department of Immunobiology, Yale University School of Medicine, 300 Cedar Street, New Haven CT 06519, USA; 2 Department of Cell Biology, Yale University School of Medicine, 300 Cedar Street, New Haven CT 06519, USA

## Abstract

PMEL is a pigment cell protein that forms physiological amyloid in melanosomes. Many amyloids and/or their oligomeric precursors are toxic, causing or contributing to severe, incurable diseases including Alzheimer’s and prion diseases. Striking similarities in intracellular formation pathways between PMEL and various pathological amyloids including Aβ and PrP^Sc^ suggest PMEL is an excellent model system to study endocytic amyloid. Learning how PMEL fibrils assemble without apparent toxicity may help developing novel therapies for amyloid diseases. Here we identify the critical PMEL domain that forms the melanosomal amyloid core (CAF). An unbiased alanine-scanning screen covering the entire region combined with quantitative electron microscopy analysis of the full set of mutants uncovers numerous essential residues. Many of these rely on aromaticity for function suggesting a role for π-stacking in melanosomal amyloid assembly. Various mutants are defective in amyloid nucleation. This extensive data set informs the first structural model of the CAF and provides insights into how the melanosomal amyloid core forms.

Amyloid fibrils are β-sheet-rich aggregates whose basic building blocks are often steric zippers^1^ or β-solenoids^2^. Their stability depends on a variety of interactions including hydrogen bonds, electrostatic interactions, hydrophobic contacts, and aromatic π-π stacking^3^,^4^. Amyloids are linked with many incurable diseases including Alzheimer’s, Parkinson’s, and prion diseases. Such diseases are dramatically gaining impact as an aging population poses new challenges to our society. Better understanding how amyloids form, how their formation is controlled, and how amyloids interact with their environment will promote the development of urgently needed novel therapies. However, amyloids are not strictly pathological structures. Many physiological amyloids have been discovered serving important functions in various organisms^5^,^6^,^7^,^8^. Because physiological amyloids do not seem to harm their cellular or tissue environment studying how they assemble may teach us how to mitigate toxicity of their pathological counterparts.

The melanocyte-specific protein PMEL (also called Pmel17 or gp100) forms physiological, pigmentation-associated amyloid^5^ and is a critical melanoma antigen^9^. In melanosomes, the protein forms a fibrillar matrix on which the UV-shielding pigment melanin is deposited^10^. Mutations in PMEL are associated with pigmentation disorders and/or impairments in eye development in various species including dogs, mice, chickens, horses, cattle, and fish^10^,^11^,^12^, strongly suggesting that PMEL has the potential to cause pigmentation aberrations and/or eye defects also in humans. Moreover, PMEL is an excellent model system to study mechanisms of intracellular amyloid formation^10^.

There are many similarities between PMEL biology and the biology of pathological amyloids. For instance, certain regulatory strategies are common among amyloids, such as the proteolytic release of a fibrillogenic peptide from a non-fibrillogenic precursor. Examples besides the PMEL core amyloid fragment (CAF)^13^,^14^ include Alzheimer’s Disease-associated Aβ^15^ and familial British/Danish dementia-associated BRI2-mutant peptides^16^. The processing of these amyloids involves an overlapping set of proteases, including proprotein convertases, α, β, and γ-secretases^15^,^16^,^17^,^18^,^19^,^20^. Fibril formation by Aβ, prion protein PrP, and PMEL can occur intracellularly inside multivesicular compartments^21^,^22^,^23^,^24^. In AA and AL amyloidosis, clinical disorders in which serum amyloid A-derived fragments and immunoglobulin light chains, respectively, accumulate as insoluble fibrils, amyloid forms in lysosomes^25^,^26^. However, unlike PMEL which remains melanosomal, in all above pathologies amyloid is eventually deposited extracellularly. While the cholesterol-rich lipid composition of intralumenal vesicles (ILVs) has been proposed to support pathological amyloid formation^27^, the transfer of PMEL to ILVs is essential to initiate fibril formation in melanosomes^28^. Moreover, low pH as found in endo-/lysosomes frequently promotes amyloid formation in both physiological and pathological systems^7^,^29^,^30^,^31^,^32^,^33^,^34^,^35^. These striking similarities indicate that studying PMEL may reveal deep insights of broad relevance for amyloid biology.

PMEL is a type I transmembrane protein. Along the secretory route, it is processed into a lumenal Mα fragment disulfide-linked to a membrane-integrated Mβ fragment^17^,^18^. In melanosomes, Mα is cleaved off the membrane^20^ and processed into an N-terminal MαN and a C-terminal MαC fragment^36^. The repeat domain (RPT) within fibril-associated MαC was originally proposed to represent the amyloid core^37^, but it is now clear that this domain can be deleted without loss of amyloidogenicity *in vitro*^14^ and *in vivo*^13^ (why the RPT domain is unlikely to represent the PMEL amyloid core is also extensively discussed in a recent review^10^). In 2009, Watt and co-workers discovered a second fibril-associated PMEL fragment of ∼8 kDa, which is liberated from MαN. They showed that it forms the amyloid core^14^ and we therefore refer to this fragment as the core amyloid fragment (CAF). Based on antibody reactivity, the CAF contains amino acids 206–220^14^ but no other sequence information is available. Knowing the amyloidogenic unit that forms the melanosomal fibrils is an essential prerequisite to understand how these fibrils are made or to reconstitute the process *in vitro*. Such *in vitro* studies may allow in the future to determine how PMEL assembly avoids toxicity in the melanosome. Unraveling the identity of the CAF has therefore remained a major goal in the field.

## Results

### Mapping domains and fragments in PMEL

**Figure 1:**
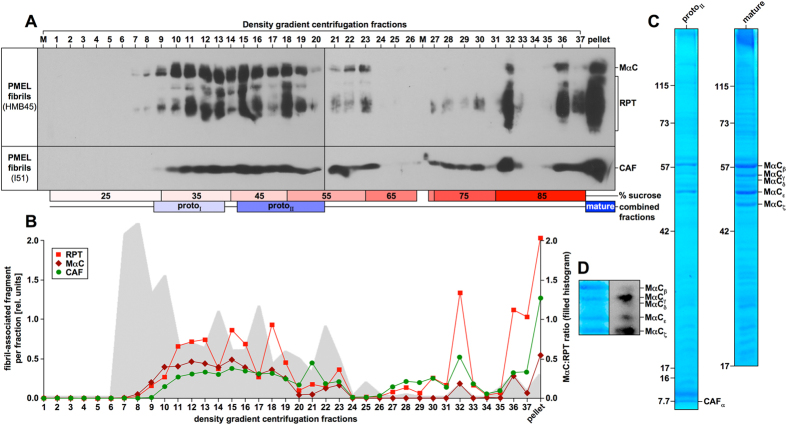
Isolation of PMEL fibrils. (**A**) PMEL fibril enrichment via velocity gradient centrifugation coupled with subsequent Triton X-100 extraction of all fractions. Triton X-100-insoluble material from each fraction was analyzed by Western blot. (**B**) Band intensities in **(A)** were determined densitometrically (*lines*). MαC:RPT ratios are shown as filled histogram. (**C**) SDS-PAGE and Coomassie Blue staining of proto_ΙΙ_ and mature fractions. (**D**) A mature fraction analyzed by Western blot and Coomassie Blue staining.

Using a velocity gradient centrifugation-based protocol we isolated melanosomal fibrils from the human melanoma cell line Mel220 stably expressing PMEL^38^. Triton X-100-lysed cellular membranes were applied to a sucrose gradient, and after fractionation each fraction was washed with Triton X-100 to remove detergent-soluble components. Fibril-enriched material included the core amyloid fragment (CAF) as well as MαC and its processed derivatives collectively called RPT. This material distributed over a large portion of the sucrose gradient (Fig. 1A). It was also found in the pellet, where besides the CAF, mostly mature RPT fragments but only relatively low levels of its precursor MαC were detected (Fig. 1A,B). Thus, the pellet largely contained fully processed fibrils, which we refer to as the “mature fraction”. In contrast, fibrillar material found along the sucrose gradient contained high levels of immature MαC relative to the mature RPT fragments. This was particularly evident in lower density fractions, and the MαC:RPT ratio steadily decreased as sucrose density in fractions increased (Fig. 1B). This incompletely processed material may represent developing protofibrils that have not yet undergone full maturation. The respective fractions were assigned to a low and a high density population, each of which was pooled, and named proto_Ι_ and proto_ΙΙ_ (Fig. 1A).

**Figure 2:**
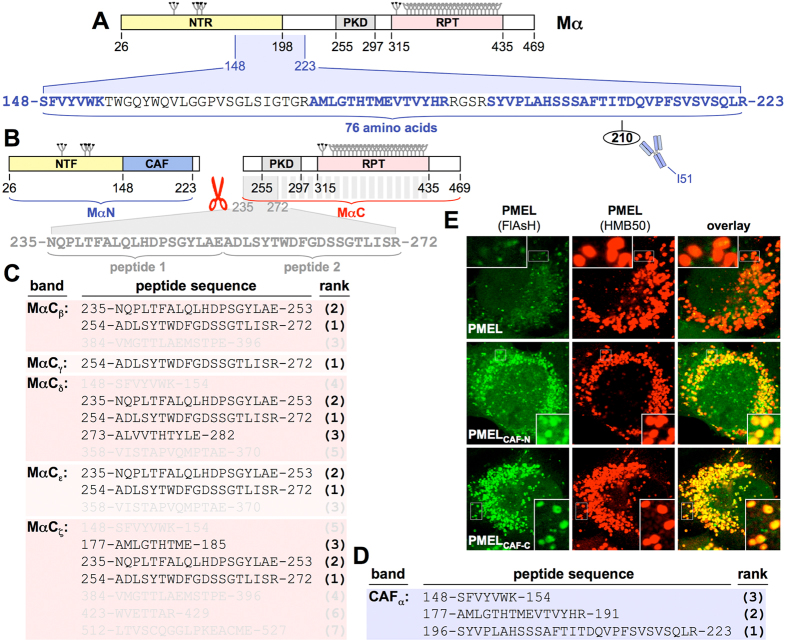
Identification of the PMEL core amyloid fragment. (**A**) The mass spectrometry-identified CAF mapped onto the Mα domain structure. The CAF largely overlaps with an N-terminal region, previously referred to as NTR^13^, but not with the PKD domain. Identified CAF-derived peptides are shown in blue. (**B**) MαN and MαC domain structure as determined by mass spectrometry. Identified MαC-derived peptides are shown in grey. (**C,D**) PMEL peptides identified by mass spectrometry in the indicated bands. High confidence peptides (score greater than identity score) shown in black. Lower confidence peptides (score lower than identity score) shown in grey. Peptides are ranked according to score (*bold brackets*). (**E**) IF analysis of FlAsH-labeled Mel220 cells stably expressing wt or tetracysteine-tagged PMEL. Antibody HMB50 labels mature fibrils.

CAF and MαC/RPT-related fragments were detected in the proto_ΙΙ_ and mature fraction. Bands were named as depicted in Fig. 1C. Low molecular weight RPT fragments were not detectable (Fig. 1C), probably due to extensive glycosylation of the repeat domain^36^ which can interfere with Coomassie labeling^39^. All five selected MαC/RPT-related bands, likely representing glycosylation isoforms and/or C-terminally truncated MαC derivatives, reacted with the PMEL-specific antibody HMB45, although to different extents (Fig. 1D). We note that differential glycosylation may affect the reactivity with Coomassie Blue and/or antibody HMB45, whose cognate epitope itself depends on sialylation^40^. Thus, it is not necessarily expected that material staining extensively with Coomassie Blue will also stain extensively with antibody HMB45. For protein identification, the indicated CAF and MαC/RPT bands were excised from the gels shown in Fig. 1C, subjected to trypsin (CAF) or trypsin/GluC digest (MαC/RPT), and analyzed by LC-MS/MS (tandem mass spectrometry). As expected, all samples contained PMEL-derived peptides (Fig. 2A–D).

Previously, the CAF had been speculated to correspond to the polycystic kidney disease (PKD) domain^14^. However, no peptides derived from this domain were found in the CAF band (CAF_α_). Rather, we detected three peptides (Fig. 2A, *blue* and Fig. 2D) spanning 76 amino acids within a more N-terminal region. This size fits well with the ∼8 kDa molecular weight of the CAF (Fig. 1C) and the respective region contains a known CAF-associated antibody-binding epitope around Thr-210 (Fig. 2A)^13^.

To confirm our mapping, we inserted into PMEL a tetracysteine tag either preceding (PMEL_CAF-N_) or following (PMEL_CAF-C_) the CAF. For both locations, FlAsH labeling of the tag highly co-localized with melanosomal amyloid (Fig. 2E) indicating its incorporation into fibrils. Thus, residues downstream of Ser-148 are indeed part of the fibrils not of the soluble PMEL N-terminus (NTF). Conversely, NTF-specific epitopes are restricted to the first 147 residues (Suppl. Fig. S1). Our mapping of the CAF is further supported by a previous study finding amyloidogenicity in a PMEL region overlapping the CAF and limited proteolysis experiments on recombinant PMEL fibrils^14^.

Cleavage of Mα separates MαN (NTF-CAF) from MαC. Thus, if the entire PKD domain lies downstream of the CAF (Fig. 2A), it should be part of MαC. To test this, we analyzed five distinct MαC/RPT bands by mass spectrometry (MαC and RPT fragments run as a poorly characterized group of bands likely representing glycosylation isoforms and/or truncated products). Not surprisingly, the highly O-glycosylated RPT domain, which represents the largest part of the fragment, rarely gave identifiable peptides. In contrast, the top identified peptides in all five MαC/RPT bands were derived from the PKD domain or even preceded it (Fig. 2B, *shown in grey* and Fig. 2C). We note that the exact borders of the PKD domain are controversial as different algorithms predict slightly different N- and C-termini (PROSITE 255–292, Superfamily 252–289, NCBI 233–295, SMART 229–311, Pfam 225–301). Nevertheless, our finding that the PKD domain is part of MαC is consistent with all these predictions. Taken together, with some remaining uncertainties about how far fragments extend beyond trypsin cleavage sites, we mapped the NTF, the CAF, and MαC to amino acids 25–147, 148–223, and 235–469, respectively.

### Identification of critical CAF residues

**Figure 3:**
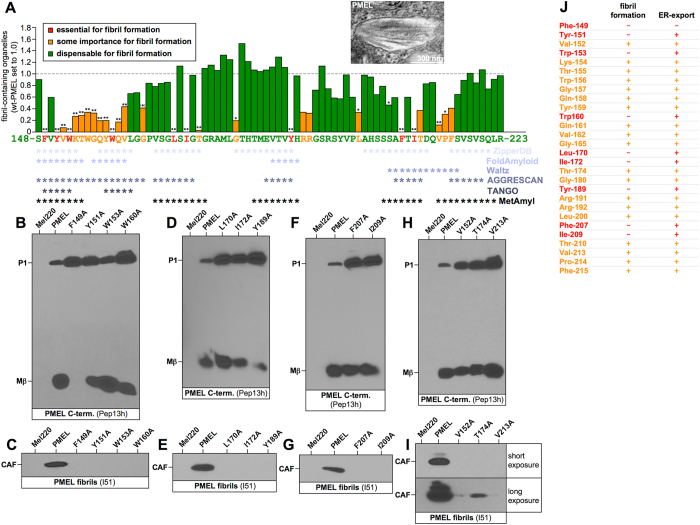
Alanine-scanning mutagenesis of the PMEL core amyloid fragment. (**A**) Quantitative EM analysis of PMEL alanine-scanning mutants. Shown is the number of fibril-containing organelles per cell [N = 15] after normalization to wt-PMEL (set to 1). The inset shows an example of a fibril-containing organelle. Essential (category 3), relevant (category 2), and largely dispensable residues (category 1) are colored in red, orange, and green, respectively. Residues predicted to be part of an amyloid-forming segment by the indicated algorithms are labeled by an asterisk. (**B–I**) Western blot analysis of SDS-lysed total membranes using PMEL-specific antibodies Pep13 h (**B,D,F,H**) and I51 (**C,E,G,I**). (**J**) Summary of category 2 and 3 residues with respect to whether they are essential for fibril formation (based on electron microscopy (Suppl. Fig. S7)) and/or ER exit (based on Mβ formation (Suppl. Fig. S2)).

To identify CAF residues that are critical for amyloid formation, we employed alanine-scanning mutagenesis. All 76 amino acids were individually exchanged to alanine (except alanines, which were exchanged to glycines and Arg-191, which was exchanged to serine) in the context of the full-length protein. Mutants were stably expressed in PMEL-free Mel220 cells and analyzed by Western blotting, immunofluorescence (IF), and quantitative electron microscopy (EM)^13^. Based on their phenotype, they were divided into three categories. Category 1 included mutants that formed fibrils at largely normal levels (Fig. 3A, *green bars*). They were efficiently exported from the ER (based on Mβ formation) (Suppl. Figs S2 and S3) and accessed melanosomes (Suppl. Fig. S6), which properly segregated away from LAMP1-positive lysosomes (Suppl. Fig. S4). The newly synthesized, mostly secretory population of these mutants (Pep13h-reactive) displayed little or no co-localization with mature fibrillar PMEL (HMB45-reactive) (Suppl. Fig. S5). This is indicative of the formation of fibrils and/or aggregates in the cell, as high epitope density on fibrils depletes HMB45 labeling from earlier compartments^13^. EM analysis confirmed the presence of fibril-containing melanosomes (Suppl. Fig. S6) at (near-) normal levels (Suppl. Fig. S7 and Fig. 3A, *green bars*). We note that along with the data of 60 novel mutants generated for this study, Fig. 3A also incorporates published quantitative EM data for 15 previously reported mutants (mutant at residues 153–162 and 194–198)^13^, which is shown for the sake of completeness and context.

Category 2 included mutants characterized by impaired but not abrogated fibril formation (<45% fibril-containing organelles per cell vs wt) (Suppl. Fig. S7 and Fig. 3A, *orange bars*). Many of these mutants (V152A, T174A, G180A, T210A, V213A, F215A) displayed poor segregation from LAMP1-containing lysosomes (Suppl. Fig. S8C) indicating misaggregation^13^. A subset of these mutants (V152A, T174A, V213A) displayed substantial co-localization between newly synthesized (Pep13h-reactive) and mature (HMB45-reactive) PMEL populations. Given previous results^13^, this points to dramatically reduced fibril formation (Suppl. Fig. S8A), although heterogeneity within cell lines was observed^13^ (*e.g.* Suppl. Fig. S8A, *compare V213A in row 4 and 5*). Despite a typically drastic reduction in cellular CAF and MαC/RPT levels (Fig. 3H,I and Suppl Fig. S3), all these mutants nevertheless gave rise to at least a few fibril-containing compartments (Fig. 3A and Suppl. Fig. S6).

Category 3 included nine complete loss-of-function mutants (F149A, Y151A, W153A, W160A, L170A, I172A, Y189A, F207A, and I209A). These formed no fibrils at all (Fig. 3A, *red bars* and Suppl. Fig. S7) and neither the CAF nor MαC/RPT fragments accumulated in the cell (Fig. 3C,E,G and Suppl. Fig. S3). In this set, only F149A was fully retained inside the endoplasmic reticulum (ER) as indicated by the absence of both Mβ and Mα (Fig. 3B and Suppl. Figs S2A and S3A), suggesting that its folding did not pass ER quality control. For I172A, Mβ (Fig. 3D and Suppl. Fig. S2C) but surprisingly no Mα (Suppl. Fig. S3C) was observed. This mutant did also not react with antibodies HMB45 (recognizing Golgi and post-Golgi PMEL) (Suppl. Fig. S8B) and HMB50 (recognizing PMEL in a conformation-sensitive manner) (Suppl. Fig. S8D), indicating that the construct is misfolded and that the Mα portion is rapidly degraded in the cell. All other loss-of-function mutants exited the ER, albeit some did so at a reduced rate (Fig. 3B,D,F) and were detected in the perinuclear region and/or early endo-/melanosomal compartments. Some of the mutants built up low levels of lysosomal aggregates (Suppl. Fig. S8D, *e.g. Y189A*), which EM suggests are non-fibrillar (Fig. 3A and Suppl. Fig. S7). In post-ER compartments, these mutants showed extensive co-localization of their newly synthesized (Pep13h-reactive) and mature (HMB45-reactive) populations, particularly in the perinuclear region (Suppl. Fig. S8B), indicating a severe reduction or a complete lack of fibrils/aggregates^13^ (see Fig. 3J for a summary of key residues that are essential for fibril formation and/or ER exit). Invariably, essential amino acids lay in regions predicted to be amyloidogenic by various prediction algorithms. This suggests a direct role of these residues in amyloid formation (Fig. 3A, *asterisks*).

### Aromatic residues are critical for PMEL amyloid formation

**Figure 4:**
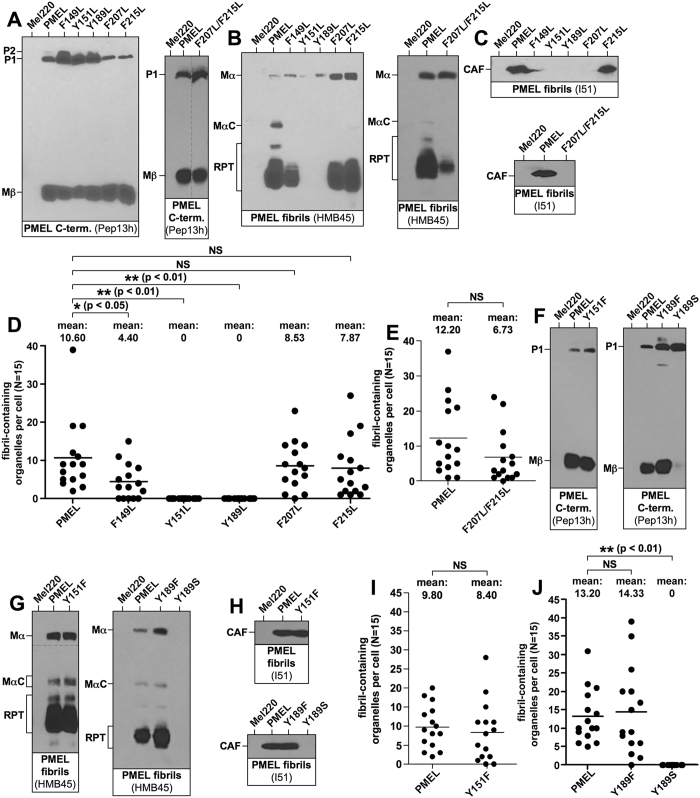
Role of aromatic amino acids in PMEL amyloid formation. (**A–C,F–H**) Western blot analysis of SDS-lysed total membranes derived from Mel220 cells stably expressing PMEL leucine (**A–C**) or phenylalanine (**F–H**) substitution mutants. PMEL-specific antibodies Pep13 h (**A,F**), HMB45 (**B,G**), and I51 (**C,H**) were used. Horizontal dashed lines separate different exposures of the same blot. Vertical dashed lines indicate positions where irrelevant lanes have been removed from the image. (**D,E,I,J**) EM analysis of Mel220 transfectants showing the number of fibril-containing organelles per cell [N = 15].

Aromatic amino acids are abundant among essential CAF residues, suggesting that aromaticity may be key for PMEL amyloid formation. To address this, we individually replaced in the context of full-length PMEL all essential aromatic residues (except previously characterized Trp-153 and Trp-160^13^) with leucine. Mutants were stably expressed in Mel220 cells and displayed normal early maturation (Fig. 4A,B).

Strikingly, the Y151L and Y189L mutations completely abrogated fibril formation, as did a Y189S replacement. No CAF (Fig. 4C,H) or MαC/RPT (Fig. 4B,G) accumulated and EM confirmed the absence of fibril-containing compartments (Fig. 4D,J). The loss-of-function phenotype was also evident by overlap of Pep13h- and HMB45-specific labeling in the perinuclear region in IF-based assays (Suppl. Fig. S9A). Thus, the mere presence of a bulky hydrophobic amino acid in positions 151 and 189 or the presence of a polar amino acid in position 189 is not sufficient to drive amyloid formation. To assess whether the specific nature of tyrosine or simple aromaticity is required in these positions, we exchanged both tyrosines individually to phenylalanine. Both mutants, Y151F and Y189F, were found to be fully functional (Fig. 4F–J and Suppl. Fig. S9A). Taken together, we conclude that it is the aromaticity, not mere hydrophobicity, in positions 151 and 189 that is essential for amyloid formation.

In contrast, Phe-149 and Phe-207, as well as Phe-215, could be replaced by leucine without completely abrogating fibril formation (Suppl. Fig. S9B). However, fibril formation was reduced for F149L (Fig. 4B–D). No such impairment was observed for F207L and F215L (Fig. 4B–D) (note that F207L is not detected by antibody I51, as Phe-207 is part of the cognate epitope). However, when both these phenylalanines were exchanged to leucine simultaneously (F207L/F215L) (Fig. 4A), fibril formation was reduced (Fig. 4B,E). This suggests that aromaticity in positions 149, 207, and 215 is also preferred, although it is not essential.

### Various CAF loss-of-function mutations impair amyloid nucleation

**Figure 5:**
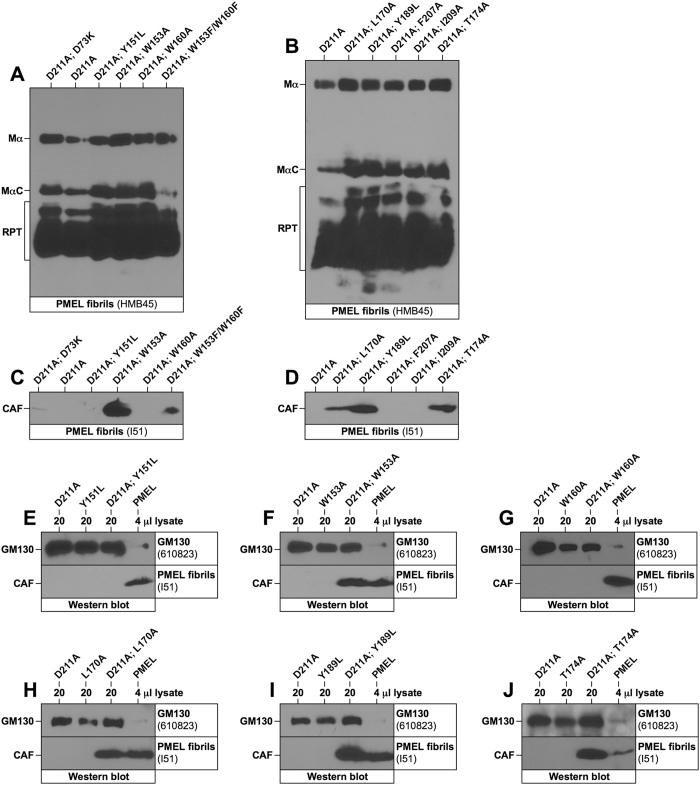
Various PMEL loss-of-function mutants are defective in amyloid nucleation. (**A–J**) Western blot analysis of SDS-lysed total membranes derived from Mel220 cells stably co-expressing the functional PMEL variant D211A with various PMEL loss-of-function mutants. PMEL-specific antibodies HMB45 (**A,B**) and I51 (**C–J**) were used. GM130 serves as loading control in (**E–J**).

Next, we asked whether CAF loss-of-function mutants act as dominant negatives, *i.e.* whether they suppress amyloid formation by co-expressed functional PMEL. For these experiments, we employed the wildtype-like, fully functional mutant D211A (Fig. 3A and Suppl. Fig. S3I), which does not react with antibody I51. Eight loss-of-function mutants (Y151L, W153A, W160A, W153F/W160F, L170A, Y189L, F207A, I209A) (Fig. 3 and ref. 13) and one mutant with severely reduced function (T174A) were individually co-expressed together with amyloid-producing PMEL-D211A (Suppl. Fig. S10A). Interestingly, none of these mutants exerted a dominant negative effect as fibril-associated MαC/RPT fragments accumulated normally even after two weeks of co-expression (Fig. 5A,B).

Next, we examined whether loss-of-function CAF subunits are incorporated into functional amyloid in above co-expression system. To this end, we exploited the fact that D211A is not recognized by antibody I51 (Suppl. Fig. S3S), which had been raised against PMEL residues 206–220^14^,^38^. Because loss-of-function mutants alone do not give rise to detectable CAF levels (Fig. 3C,E,G,I) and the D211A-CAF is not visible in I51-labeled Western blots, co-expression of the constructs allows to examine whether mutant subunits can be stabilized *in trans, i.e.* whether they co-assemble into a common amyloid. Strikingly, this appears to be the case for mutants W153A, W153F/W160F, L170A, Y189L, and T174A (Fig. 5C,D,F,H–J). No argument can be made for F207A and I209A (Fig. 5D), as these constructs themselves do not react well with antibody I51.

That various loss-of-function mutants are stabilized *in trans* by functional PMEL without disrupting amyloid formation (Fig. 5A,B) suggests they properly incorporate into nascent fibrils. This argues against severe irreversible folding issues and rather points to a possible problem in the nucleation step, which can be overcome if another construct initiates amyloid formation in the cell. Interestingly, only two PMEL mutants could not be stabilized *in trans*: Y151L (Fig. 5C,E) and W160A (Fig. 5C,G). This indicates that Tyr-151 and Trp-160 play a crucial role distinct from other essential amino acids.

### Conserved regions of high predicted amyloidogenicity decorate the four rungs of a β-solenoid CAF structural model

**Figure 6:**
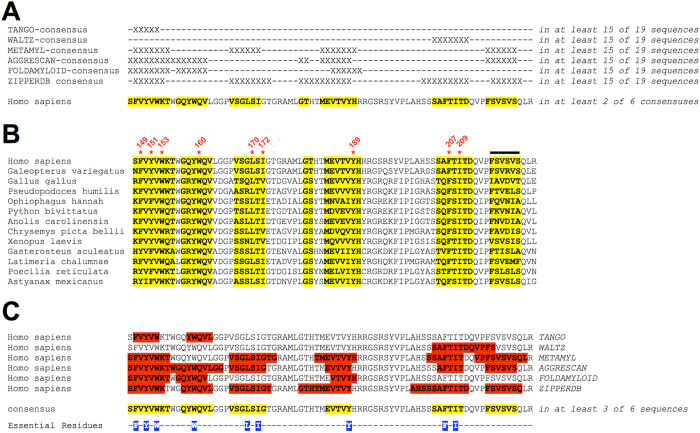
Conservation of amyloidogenic segments in PMEL. (**A**) Prediction of amyloidogenic blocks in the human CAF as well as in the corresponding CAF sequence derived from 18 other species. These 18 other species include three more mammals, four reptiles, one amphibian, three birds, and seven fish. X indicates positions predicted to be part of an amyloidogenic segment in at least 15 out of the 19 species. A consensus sequence of predicted amyloidogenic segments was constructed for each of the six algorithms (*i.e.* the set of residues labeled X) (Suppl. Fig. S10B). Then, based on the six consensus sequences a super-consensus sequence was determined (*i.e.* the set of residues predicted to be part of an amyloidogenic segment in at least two consensus sequences) and mapped onto the human CAF sequence (*shown in yellow*). (**B**) The super-consensus sequence mapped onto an alignment of selected species including mammals, reptiles, birds, amphibians, and fish (*shown in yellow*). Human key residues are numbered. The black line indicates a segment that shows significant variability in sequence, but is nevertheless consistently predicted to be amyloidogenic. (**C**) Amyloid forming segments predicted for the human CAF sequence are highlighted in red. The human consensus sequence (*i.e.* the set of residues predicted to be part of an amyloidogenic segment by at least three algorithms) is mapped onto the human CAF sequence (*shown in yellow*). Essential residues are highlighted in blue.

Six algorithms were used to identify aggregation-prone or amyloidogenic segments within the human CAF or the corresponding sequence from various other species: TANGO^41^, WALTZ^42^, METAMYL^43^, AGGRESCAN^44^, FOLDAMYLOID^45^, and ZIPPERDB^46^. For each algorithm a consensus sequence was generated (Suppl. Fig. S10B). Based on these consensus sequences, a super-consensus was built and mapped onto the human CAF (Fig. 6A,B, *yellow*). Separately, a “human consensus sequence” was constructed based on the human CAF sequence alone (Fig. 6C, *yellow*). The super-consensus and the human consensus sequence are not only highly similar but also contain all essential residues (Fig. 6C, *blue*), strongly suggesting that functional amyloidogenicity of sequence blocks is conserved. Remarkably, this is true even at positions where significant sequence variability exists among different species (Fig. 6B, segment indicated by black line). Most key residues, including Trp-153, Trp-160, Leu-170, Tyr-189, and Phe-207 are absolutely conserved. Positions 149 and 151 are invariably occupied by tyrosine or phenylalanine. Positions 172 and 209 are invariably occupied by isoleucine, valine, or leucine (Fig. 6B).

**Figure 7:**
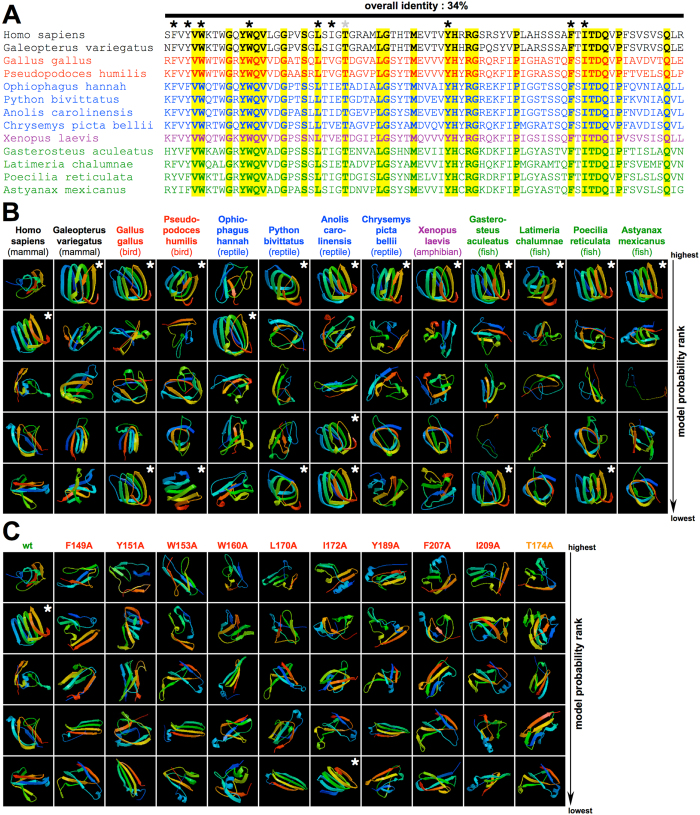
Structural modeling of the PMEL core amyloid fragment. (**A**) Alignment of selected CAF sequences derived from mammals (*black*), birds (*red*), reptiles (*blue*), amphibians (*purple*), and fish (*green*). Essential residues are indicated with a black asterisk. Identity is highlighted in yellow. (**B**) ROBETTA structure prediction for the CAF sequences from **(A)**. The five likeliest models are ranked from top to bottom. A white asterisk indicates a β-solenoid structure. (**C**) ROBETTA predictions for the human CAF sequence, in which essential residues have been substituted by alanine.

**Figure 8:**
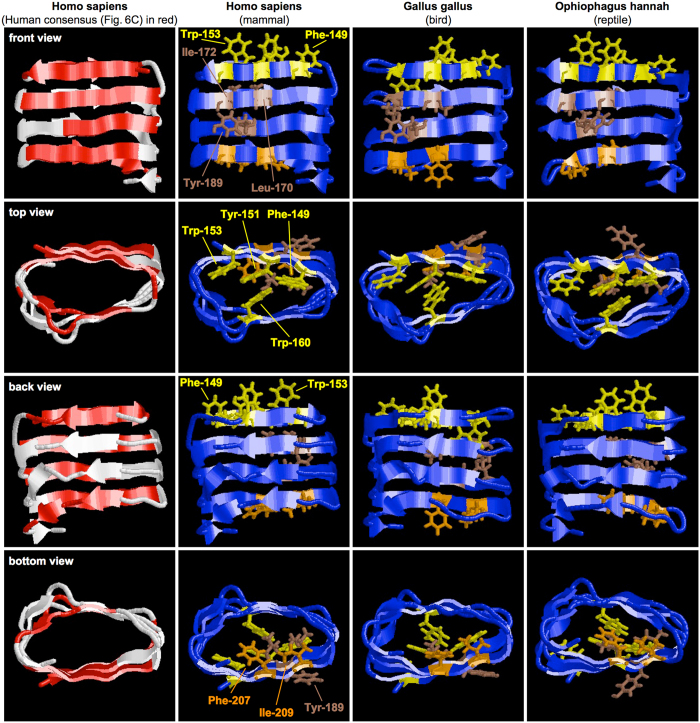
A β-solenoid structural model for the PMEL core amyloid fragment. ROBETTA-predicted β-solenoid model for human, highest ranking chicken, and cobra (see Materials & Methods) CAF. The “human consensus sequence” is mapped in red onto the structure (*column 1*). In columns 2–4, essential residues are highlighted as sticks (yellow in top rung, brown in inner rungs, orange in bottom rung). All images were created with RasWin 2.7.5.2.

To get insights into a possible structure for the CAF, we used the comparative modeling algorithm on the ROBETTA server^47^. Structures were predicted for the human CAF as well as for the corresponding sequence derived from various species including other mammals, birds, reptiles, amphibians, and fish (Fig. 7B). The ROBETTA server calculates the five likeliest structure models and ranks these according to their probability (Fig. 7B). Among these results, a group of highly related structures stood out corresponding to a β-solenoid, a four coil right-handed β-helix (Figs 7B and 8). This β-solenoid structure was often the top-ranked model, while it was the second highest ranking model for the human CAF (Fig. 7B, *white asterisks*). For several species more than one β-solenoid structure clustered among the five likeliest models and these were all highly related (Fig. 7B, *white asterisks*). Although the overall sequence identity was only 34% (Fig. 7A), predicted β-solenoid structures were remarkably similar across species (Fig. 8). In contrast, all but one of the alanine loss-of-function mutations identified in our study (Fig. 3C,E,G) completely abrogated the prediction of a β-solenoid structure for the human CAF when inserted into the submitted sequence (Fig. 7C, *white asterisks*). The only exception was I172A, which – as discussed above – may cause early Mα degradation rather than affecting amyloid formation directly (Fig. 7C).

In the β-solenoid structure predicted for the human CAF (Fig. 8, *column 1*) a front face is packed against a back face at a distance of ~9.5 Å, establishing a dry interface excluding water (Fig. 8, *row 2*). The front face consists of four continuous β-strands each six to eight amino acids long, stacking atop of each other, individual strands being separated from each other by ~ 4.7 Å (Fig. 8, *row 1*). The back face contains shorter discontinuous β-strand segments, which are also stacked (Fig. 8, *row 3*). When the “human consensus sequence” from Fig. 6C is mapped onto the model, it covers almost the entire front face as well as significant portions of the top and bottom rung of the back face (Fig. 8, *red in column 1*). All essential residues except Tyr-189 point inwards into the β-helix and all essential residues except Trp-160 localize to the front face. Strikingly, in this arrangement Tyr-151 and Trp-160 are positioned vis-à-vis in the top rung at a distance of ~3.5 Å, seemingly π-stacking against each other. Thus, these residues may critically stabilize the β-solenoid or drive its formation. Our biochemical results in Fig. 5E,G had pointed to a particularly crucial role for Tyr-151 and Trp-160 in PMEL amyloid formation. Our structural model may underpin this observation mechanistically.

## Discussion

N-terminal PMEL fragments encompassing the CAF are amyloidogenic *in vitro*, as are subfragments containing only part of the domain (aa 25–205, aa 201–314)^14^. Thus, multiple independent amyloidogenic determinants could be present, which is in line with our prediction of various amyloidogenic motifs scattered throughout the CAF sequence (Fig. 6C). Similar observations have been made with other fibril-forming molecules including Aβ, where *e.g.* peptides aa 11–25^48^ and aa 37–42^49^ independently form amyloid *in vitro*. All essential residues localize to predicted amyloidogenic blocks and many are aromatic (Fig. 6). Aromatic amino acids are common and frequently favored in amyloids^50^ due to their hydrophobicity, β-strand propensity, and potential to engage in π-stacking interactions^51^,^52^. Moreover, aromatic residues may be required to form optimal steric zipper interfaces^53^ and they promote fibril fragmentation in yeast prions^54^.

A key role for π-stacking of aromatic amino acids has been established in some amyloid systems^51^,^52^, while mere hydrophobicity/β-strand propensity is important in others^55^. Substitution of critical aromatic residues with bulky hydrophobic leucine often drastically reduces amyloid formation^53^,^56^,^57^,^58^. Further, in some systems early stages of amyloid assembly may depend on different chemical aspects of aromatic residues (*e.g.* hydrophobicity, β-sheet propensity) than late stages (*e.g.* π-stacking)^59^. Finally, aromatic residues have been implicated in affecting fibril morphology and properties^60^.

In PMEL we identify a spectrum of essential aromatic residues with distinct requirements for amino acid chemical nature. Tryptophans (Trp-153, Trp-160) cannot even be replaced by phenylalanine without severely impairing fibril formation^13^ (Figs 3A,C and 5F,G). Tyrosines (Tyr-151, Tyr-189) are more flexible in this respect (Fig. 4F–J). However, their replacement with leucine, which has a similar molecular volume, hydrophobicity, and β-sheet propensity as phenylalanine^51^, causes a complete loss of fibrils (Fig. 4A–D). Thus, Tyr-151 and Tyr-189 may participate in specific aromatic interactions that cannot be supported by mere hydrophobicity. Tyrosine also has a polar character via its hydroxyl group, but at least in the case of Tyr-189, this residue cannot be functionally replaced by serine (Tyr-151 was not tested).

Among essential aromatic residues, phenylalanines (Phe-149, Phe-207) seem the most flexible with respect to aromaticity. All of them can be replaced with leucines without complete loss-of-function. However, F149L displays reduced fibril formation, as does F207L/F215L (Fig. 4A–D), suggesting that aromaticity in positions 149, 207, and 215 is also preferred. Importantly, the aromaticity of all six essential aromatic residues is absolutely conserved throughout evolution (Fig. 7A), pointing to a key role of these amino acids in the protein’s function.

Although any model of how the CAF may fold must be tentative until solid structural data becomes available, our predicted model (Fig. 8) has many attractive features and is fully consistent with experimental data. PMEL fibril formation is functionally conserved down to zebrafish^10^, but the overall sequence identity of the CAF is only 34% (Fig. 7A). Despite this, for the majority of examined CAF sequences a β-solenoid was among the five likeliest ROBETTA-predicted models, typically representing the top hit (Fig. 7B). For several species two or more related β-solenoid structures clustered among the five likeliest models, suggesting its preference (Fig. 7B, *white asterisks*). Among the five structures predicted for the human CAF, the β-solenoid was the only one that was also repeatedly predicted for distantly related species. When loss-of-function CAF sequences were submitted to ROBETTA, no β-solenoid structure was predicted (Fig. 7C) except for I172A. As discussed above, I172A is a special case, in which loss-of-function is probably a result of early Mα degradation (Suppl. Fig. S3C). Thus, ROBETTA significantly favors a β-solenoid structure for the CAF and, consistent with our alanine-scanning data (Fig. 3), the prediction of this structure requires sequence integrity at positions our study shows are essential.

β-solenoids have been proposed to form the basic building blocks for various amyloids including prions^6^,^8^,^61^,^62^,^63^,^64^,^65^. Strikingly, the scrapie prion protein PrP^Sc^ forms amyloid fibrils composed of β-solenoid subunits containing four rungs, exactly what we predict for the CAF^66^. Thus, the CAF and PrP^Sc^ may share a remarkably similar structural architecture, which has important consequences for the nucleation mechanism^66^. Stacking β-solenoid subunits could give rise to a cross-β structure in which β-strands run perpendicular to the fibril axis enclosing a dry, largely hydrophobic core. Consistent with their hydrophobic nature, eight of nine essential residues point inwards into this core, packing the β-sheets of the front and back face against each other. Four essential aromatic amino acids are located in the top rung, where they appear to form a π-stacking system potentially involving Trp-153, Trp-160, Tyr-151, and Phe-149. Tyr-151 is the central residue in this system and could act as an organizing hub coordinating various interactions. Consistent with this view, the aromaticity of Tyr-151 is essential for PMEL fibril formation (Fig. 4). Specifically, our structural model features Tyr-151 π-stacking against Trp-160 in human and non-human CAF models (Fig. 8), an interaction that could stabilize the β-solenoid or drive its formation. This interaction could help establishing and stabilizing the top rung (Fig. 8), given our mutagenesis analysis the most critical CAF region (Fig. 3A), providing a template for the next incoming β-strand. A key role for these two residues is also suggested by our biochemical experiments showing that Y151L and W160A are the only mutants that cannot be rescued by cross-nucleation (Fig. 5E,G).

Relevant (category 2) and essential (category 3) residues highly cluster at the N-terminus (87% of first 15 aa) and to a lesser extent at the C-terminus (35% of last 17 aa) of the CAF, whereas the middle 44 aa segment contains only relatively few (20%) (Fig. 3A). Thus, such residues massively dominate on the strongly aromatic top rung and are also enriched on the bottom rung of the predicted β-solenoid. This fits well with the functional conservation of amyloidogenic motifs, which also map most extensively to the top and bottom rung, while in the two inner rungs such motifs are limited to the front face (Fig. 8). Accordingly, the evolutionary conservation is also significantly higher at the N- and C-termini than in the middle segment of the CAF. We note that if the predicted β-solenoid were stacked into protofilaments, the top and bottom rungs would represent the subunit-subunit interaction interface. This might explain why these regions display stronger conservation and contain so many functionally critical residues.

Tyr-189 is the only essential residue, together with several category 2 residues, that consistently points outwards from the predicted β-solenoid (Fig. 8) and could function in the assembly of protofilaments into fibrils. Interestingly, many of these residues distribute around one of the stacked turn regions. Three of them are basic (Lys-154, Arg-191, Arg-192) and their clustering may cause a local positive charge. We note that a role for Arg-191 in lateral fibril assembly had been suspected earlier due to the disorganization of R191S fibrils^38^. Moreover, a local positive charge could mediate interactions with negatively charged phospholipid headgroups on ILVs. As lipids can accelerate the formation of other amyloids including α-synuclein^67^ and Aβ^68^, such interactions could promote CAF nucleation. Hydrophobic regions could be sequestered by association with membrane lipids to free up critical amyloidogenic residues, allowing them to stabilize the hypothetical β-solenoid. This could also potentially explain how the CAF segment is prevented from forming amyloid before accessing melanosomes. In line with such scenarios, the transfer of PMEL to ILVs in a CD63- and apolipoprotein E-dependent manner is essential for amyloidogenesis^28^,^69^ and apolipoprotein E has been suggested to act as a docking platform for PMEL on the ILVs^65^. Once activated, the CAF could potentially even seed amyloid formation by other PMEL domains, such as the PKD domain or the RPT domain, which may contain some amyloidogenic potential as well^14^,^37^. This could explain the insolubility and fibril-association of MαC as well as the fact that MαC can apparently be stabilized by CAF amyloid *in trans*^13^.

Surprisingly, many loss-of-function CAF mutants appear to become functional when co-expressed with wildtype (-like) PMEL (Fig. 5F,H–J). This strongly suggests that the respective constructs are not grossly misfolded or mistargeted. Their stabilization *in trans* (Fig. 5F,H–J) likely reflects proper incorporation into nascent amyloid. Importantly, this incorporation does not appear to “cap” fibrils with subunits not permitting further extension, as this should disrupt fibril growth in a dominant negative manner (Fig. 5A,B). Hence, the respective mutants largely retain their amyloidogenic potential, but this potential is only unlocked in the context of concomitant functional nucleation. Thus, the respective residues may mediate nucleation, *e.g.* through structurally stabilizing fibril seeds.

## Material and Methods

### Cell lines and cell culture

LG2-MEL-220 (Mel220), a human PMEL-deficient melanoma cell line^70^, was grown in IMDM (Sigma)/10% FCS (HyClone) containing non-essential amino acids (Gibco), GlutaMax (Gibco) and penicillin/streptomycin (Gibco). PMEL transfectants were grown in medium additionally containing 2 mg/ml G418 (Gibco).

### Vector constructs and PMEL expression

PMEL in pBMN-IRES-neo^38^ served as template for QuikChange mutagenesis using primer pairs listed in Table S1. Vectors were sequenced before retroviral transduction^13^ into Mel220 cells and selection with 2 mg/ml G418 (Gibco).

### Antibodies

Pep13h^21^ and ab52058 (Abcam) recognize the PMEL C-terminus. I51 recognizes the PMEL CAF^14^. HMB50 recognizes an epitope within PMEL aa 209–254^38^,^71^,^72^. HMB45 (NeoMarkers) recognizes the sialylated RPT domain^40^. EP4863(2) (Abcam), 7E3 (Abcam), and E-7 (Santa Cruz) recognize the PMEL NTF^13^. H4A3 (Abcam) recognizes LAMP1. 610823 (BD) recognizes GM130. HRP- and fluorophore-labeled secondary antibodies were purchased from Jackson ImmunoResearch and Molecular Probes.

### Immunofluorescence

IF was performed as described^18^. Briefly, Mel220 transfectants were permeabilized and stained in PBS/0.5% BSA/0.5% saponin for 1 h with primary followed by Alexa647/488-conjugated secondary antibodies at 1:100. H4A3 and HMB45 were used at 1:25 and 1:50. Cells were mounted in ProLong Gold reagent (Invitrogen). FlAsH labeling was performed as described^73^. Images were acquired using a Leica TCS SP2 or SP8 confocal microscope (Leica Microsystems) at 63x magnification.

### Western blotting

Total membranes were prepared as described^74^. Briefly, 5 × 10^6^ cells were resuspended in 1 ml 10 mM Tris-HCl pH 7.4 containing protease inhibitor (Complete, Roche) and incubated for 10 min on ice. Lysed cells were Dounce homogenized with 30 strokes (“tight” pestle) and centrifuged at 800 g/4 °C/10 min. The resulting supernatant was spun at 45,000 rpm/4 °C using the TLA-55 rotor in a Beckman Optima TL ultracentrifuge. The pellet was lysed in PBS/1% SDS/1% β-mercaptoethanol for 10 min at RT followed by 10 min at 95 °C and subjected to SDS-PAGE. Western blotting was carried out as described^75^.

### Velocity gradient centrifugation

Mel220 cells expressing PMEL were harvested from 10 confluent 175 cm^2^ tissue culture flasks and resuspended in 50 ml PBS. Cytochalasin D (Sigma Aldrich) and nocodazole (Sigma Aldrich) were added to final concentrations of 2.5 μg/ml and 10 μM, respectively, and incubated at 37 °C under slow shaking for 30 min. Next, cells were resuspended in 12 ml 10 mM Tris-HCl pH 7.4 containing protease inhibitor (Complete, Roche) and incubated for 10 min at 4 °C. Lysed cells were divided into 1 ml portions and total membranes were prepared as described above. After rinsing membrane pellets with 500 μl high-salt buffer (prepared by mixing 2 ml 10x PBS with 18 ml 2 M NaCl + protease inhibitor (Complete, Roche)) the material was re-combined and incubated in 8 ml high-salt buffer for 45 min at 4 °C. Membranes were collected by ultracentrifugation as described above, rinsed twice with PBS and stored at −80 °C.

Membranes were lysed in 1.8 ml PBS/2% Triton X-100 for 2 hrs at 4 °C before 85% sucrose was added to a final concentration of 25%. Next, a sucrose step gradient was prepared in a Beckman polyallomer 14 × 89 mm ultracentrifugation tube from bottom to top consisting of each 1.4 ml of 85%, 75%, 65%, 55%, 45%, and 35% sucrose (all in Tris-Cl-EDTA (50 mM Tris pH 7.4/200 mM NaCl/1 mM EDTA)). The topmost layer was overlaid with the membrane lysate and spun for 2 hrs at 39.000 rpm and 4 °C in the Beckman Optima L-90K ultracentrifuge using the SW41 Ti rotor (slow acceleration, no break). After the run, the gradient was harvested from the top in 300 μl portions, each of which were added to 900 μl Tris-Cl-EDTA. The pellet was resuspended in 1.2 ml Tris-Cl-EDTA. All samples were incubated overnight on the rotator at 4 °C. Following this, the samples were spun at 45,000 rpm and 4 °C using the TLA-55 rotor in a Beckman Optima TL ultracentrifuge and the supernatant was discarded. Pellets were extracted in 1 ml PBS/Triton X-100 + protease inhibitor (Complete, Roche) for 1.5 hrs at 4 °C, spun again at 45.000 rpm, and resuspended in 200 μl PBS and frozen at −80 °C.

### Mass spectrometry

To identify the CAF and MαC, “proto_II_” and “mature” fibril fractions were separated by SDS-PAGE, stained with Coomassie Blue, and bands labeled in Fig. 1C were excised for mass spectrometry analysis at the Yale W. M. Keck Foundation Biotechnology Resource Laboratory. LC-MS/MS data were acquired after digestion with trypsin (CAF_α_) or trypsin/Glu-C (MαC derivatives). Peptides were separated on a Waters nanoACQUITY column (75 μm × 250 mm) followed by MS analysis on an LTQ Orbitrap XL (CAF_α_, MαC_β_, MαC_γ_) or Orbitrap Elite (MαC_δ_, MαC_ε_, MαC_ζ_) mass spectrometer. Protein identification was performed with the Mascot algorithm.

### Assembly of the cobra CAF sequence

The Ophiophagus hannah CAF sequence used in our study is based on GenBank protein entry ETE74109.1. However, this predicted protein contains a highly unusual 18 aa deletion at the CAF C-terminus (corresponding to 212-QVPFSVSVSQLRALDGGN-229 in the human CAF) not found in any other species including other snakes. As the deletion is located at an exon-exon boundary, we suspected that it may be an artifact caused by incorrect prediction of a splice acceptor site. Thus, we obtained the genomic sequence containing cobra PMEL (GenBank AZIM01000004.1) and submitted a fragment containing the relevant region to the NetGene2 server^76^. NetGene2 predicted the expected donor splice site (AGCATCACTG^GTAAATATTA) (score 0.71), but, importantly, also an acceptor splice site (CCTTTCCCAG^ACCAGATCCC) (score 0.97) with very high confidence. Such splicing would result in a fully conventional CAF sequence without the unusual deletion: 207-TSSQFSITDQIPFQVNIAQLL*DTDGTDSRFVR-238 (residues deleted in GenBank entry ETE74109.1 shown in bold, asterisk indicates most C-terminal CAF residue) (see also Fig. 7A). This is completely identical to the Brown Spotted Pit Viper sequence (GenBank XP_015671811.1) and fully collinear with regular CAFs). In contrast, NetGene2 did not predict a splice acceptor site that would lead to the deletion in the published sequence. Thus, we believe that the unusual deletion in cobra PMEL is an artifact and the correct sequence is completely conventional. We use this conventional sequence for our studies in Figs 7 and 8.

### Electron microscopy

For conventional Epon embedding, cells were fixed in 2.5% glutaraldehyde/2% sucrose in 0.1 M sodium cacodylate buffer pH 7.4 (NaCaCo buffer) for 30 min at RT followed by 30 min at 4 °C. Subsequently, cells were rinsed with NaCaCo buffer and further processed as described^38^.

Samples were viewed using an FEI Tecnai Biotwin TEM at 80 Kv. Images were collected using Morada CCD and iTEM (Olympus) software.

Epon-embedded EM samples were first inspected to qualitatively determine whether the respective Mel220 transfectant formed fibril-containing melanosomes. To quantify fibril formation, we then counted fibril-containing organelles in 15 arbitrarily chosen cells in one view field. Each count was performed once and the respective means are indicated in Figs 3A and 4D,E,I,J, and Suppl. Fig. S7. A Student’s two-tailed t-test (Y151F and F207L/215 L) or a one-way ANOVA with Dunnett’s post test (all others) was used to determine whether means are statistically different from the wt-PMEL sample. Asterisks in respective figures indicate statistical significance, but are shown in brackets for construct G165A where only Dunnett’s post test but not the one-way ANOVA indicated statistical significance.

## Additional Information

**How to cite this article:** Hee, J. S. *et al*. Melanosomal formation of PMEL core amyloid is driven by aromatic residues. *Sci. Rep.* **7**, 44064; doi: 10.1038/srep44064 (2017).

**Publisher's note:** Springer Nature remains neutral with regard to jurisdictional claims in published maps and institutional affiliations.

## Supplementary Material

Supplementary Material
