# Supplementary material for: Melanosomal formation of PMEL core amyloid is driven by aromatic residues
Source: Sci Rep. 2017 Mar 8;7:44064. doi: 10.1038/srep44064 (PMC5341037; doi:10.1038/srep44064)
Supplement: Supplementary Material [file srep44064-s1.pdf]

Supplementary Material

Melanosomal formation of PMEL core amyloid is driven by aromatic residues

Jia Shee Hee [a,1], Susan M. Mitchell [a,1], Xinran Liu [b], and Ralf M. Leonhardt [a,*]

From the [a] Department of Immunobiology and the [b] Department of Cell Biology, Yale University School of Medicine, 300 Cedar Street, New Haven CT 06519, USA

[1] equal contribution

[*] Address correspondence to: Ralf M. Leonhardt, Ph.D., Yale University School of Medicine, Department of Immunobiology, 300 Cedar Street, TAC S669/670, New Haven, CT 06519-1612, USA, Tel. (203)-785-5042, Fax. (203)-785-4461; E-mail: Ralf.Leonhardt@yale.edu

Supplementary Table S1, ***PMEL-specific primers used for Quikchange mutagenesis***

S148A	5′-GG TCT CAG AAG AGA GCC TTT GTA TAC GTC TGG AAG ACC TGG-3′<br>5′-CCA GGT CTT CCA GAC GTA TAC AAA GGC TCT CTT CTG AGA CC-3′
F149A	5′-CT CAG AAG AGA AGC GCT GTT TAT GTC TGG AAG ACC-3′<br>5′-GGT CTT CCA GAC ATA AAC AGC GCT TCT CTT CTG AG-3′
V150A	5′-CT CAG AAG AGA AGC TTT GCA TAT GTC TGG AAG ACC-3′<br>5′-GGT CTT CCA GAC ATA TGC AAA GCT TCT CTT CTG AG-3′
Y151A	5′-CT CAG AAG AGA AGT TTT GTT GCT GTC TGG AAG ACC-3′<br>5′-GGT CTT CCA GAC AGC AAC AAA ACT TCT CTT CTG AG-3′
V152A	5′-CAG AAG AGA AGC TTT GTT TAC GCG TGG AAG ACC TGG G-3′<br>5′-C CCA GGT CTT CCA CGC GTA AAC AAA GCT TCT CTT CTG-3′
L163A	5′-C CAA TAC TGG CAA GTT GCC GGC GGC CCA GTG TCT GG-3′<br>5′-CC AGA CAC TGG GCC GCC GGC AAC TTG CCA GTA TTG G-3′
G164A	5′-C TGG CAA GTT CTA GCC GGC CCA GTG TCT GG-3′<br>5′-CC AGA CAC TGG GCC GGC TAG AAC TTG CCA G-3′
G165A	5′-GG CAA GTT CTA GGC GCC CCA GTG TCT GGG C-3′<br>5′-G CCC AGA CAC TGG GGC GCC TAG AAC TTG CC-3′
P166A	5′-G CAA GTT CTA GGG GGC GCC GTG TCT GGG CTG AGC-3′<br>5′-GCT CAG CCC AGA CAC GGC GCC CCC TAG AAC TTG C-3′
V167A	5′-GG CAA GTT CTA GGA GGC CCA GCG TCT GGG CTG AGC-3′<br>5′-GCT CAG CCC AGA CGC TGG GCC TCC TAG AAC TTG CC-3′
S168A	5′-GG CAA GTT CTA GGA GGC CCA GTG GCT GGG CTG AGC ATT GG-3′<br>5′-CC AAT GCT CAG CCC AGC CAC TGG GCC TCC TAG AAC TTG CC-3′
G169A	5′-GGG GGC CCA GTG AGC GCG CTG AGC ATT GGG-3′<br>5′-CCC AAT GCT CAG CGC GCT CAC TGG GCC CCC-3′
L170A	5′-GC CCA GTG TCT GGC GCC AGC ATT GGG ACA GG-3′<br>5′-CC TGT CCC AAT GCT GGC GCC AGA CAC TGG GC-3′
S171A	5′-CCA GTG TCT GGG CTG GCC ATT GGG ACA GGC-3′<br>5′-GCC TGT CCC AAT GGC CAG CCC AGA CAC TGG-3′
I172A	5′-G TCT GGG CTG AGC GCT GGG ACA GGC AGG-3′<br>5′-CCT GCC TGT CCC AGC GCT CAG CCC AGA C-3′
G173A	5′-CCA GTG TCT GGG CTG TCG ATC GCG ACA GGC AGG GCA ATG C-3′<br>5′-G CAT TGC CCT GCC TGT CGC GAT CGA CAG CCC AGA CAC TGG-3′
T174A	5′-G CTG AGC ATT GGG GCC GGC AGG GCA ATG C-3′<br>5′-G CAT TGC CCT GCC GGC CCC AAT GCT CAG C-3′
G175A	5′-G AGC ATT GGG ACA GCG CGC GCA ATG CTG GGC AC-3′<br>5′-GT GCC CAG CAT TGC GCG CGC TGT CCC AAT GCT C-3′
R176A	5′-GC ATT GGG ACA GGC GCC GCA ATG CTG GGC AC-3′<br>5′-GT GCC CAG CAT TGC GGC GCC TGT CCC AAT GC-3′
A177G	5′-CTG AGC ATT GGG ACC GGC CGG GGA ATG CTG GGC ACA C-3′<br>5′-G TGT GCC CAG CAT TCC CCG GCC GGT CCC AAT GCT CAG-3′
M178A	5′-GG ACA GGC AGG GCA GCG CTG GGC ACA CAC-3′<br>5′-GTG TGT GCC CAG CGC TGC CCT GCC TGT CC-3′
L179A	5′-CA GGC AGG GCA ATG GCG GGT ACC CAC ACC ATG GAA G-3′<br>5′-C TTC CAT GGT GTG GGT ACC CGC CAT TGC CCT GCC TG-3′
G180A	5′-GC AGG GCA ATG CTG GCC ACA CAC ACC ATG G-3′<br>5′-C CAT GGT GTG TGT GGC CAG CAT TGC CCT GC-3′
T181A	5′-G GCA ATG CTG GGC GCC CAC ACC ATG GAA G-3′<br>5′-C TTC CAT GGT GTG GGC GCC CAG CAT TGC C-3′
H182A	5′-GG GCA ATG CTG GGT ACC GCC ACC ATG GAA GTG-3′<br>5′-CAC TTC CAT GGT GGC GGT ACC CAG CAT TGC CC-3′
T183A	5′-GG GCA ATG CTG GGT ACC CAC GCC ATG GAA GTG AC-3′<br>5′-GT CAC TTC CAT GGC GTG GGT ACC CAG CAT TGC CC-3′
M184A	5′-G GGC ACA CAC ACC GCG GAA GTG ACT GTC-3′<br>5′-GAC AGT CAC TTC CGC GGT GTG TGT GCC C-3′
E185A	5′-G CTG GGC ACA CAC ACA ATG GCA GTG ACT GTC TAC C-3′<br>5′-G GTA GAC AGT CAC TGC CAT TGT GTG TGT GCC CAG C-3′
V186A	5′-G CTG GGC ACA CAC ACA ATG GAA GCG ACT GTC TAC CAT CG-3′<br>5′-CG ATG GTA GAC AGT CGC TTC CAT TGT GTG TGT GCC CAG C-3′
T187A	5′-C ACC ATG GAA GTG GCT GTA TAC CAT CGC CGG G-3′<br>5′-C CCG GCG ATG GTA TAC AGC CAC TTC CAT GGT G-3′
V188A	5′-CC ATG GAA GTG ACT GCC TAC CAT AGG CGG GGA TCC CGG-3′<br>5′-CCG GGA TCC CCG CCT ATG GTA GGC AGT CAC TTC CAT GG-3′
Y189A	5′-CC ATG GAA GTG ACT GTT GCG CAT CGC CGG GGA TCC CGG-3′<br>5′-CCG GGA TCC CCG GCG ATG CGC AAC AGT CAC TTC CAT GG-3′
H190A	5'-GTG ACT GTC TAC GCT AGG CGG GGA TCC CGG-3′<br>5′-CCG GGA TCC CCG CCT AGC GTA GAC AGT CAC-3′
R192A	5′-CT GTC TAC CAT CGC GCG GGT TCC CGG AGC TAT G-3′<br>5′-C ATA GCT CCG GGA ACC CGC GCG ATG GTA GAC AG-3′
G193A	5′-C TAC CAT CGC CGG GCA TCC CGG AGC TAT GTG C-3′<br>5′-G CAC ATA GCT CCG GGA TGC CCG GCG ATG GTA G-3′

P199A	5′-CC CGG AGC TAT GTA GCG CTT GCT CAT TCC AGC-3′<br>5′-GCT GGA ATG AGC AAG CGC TAC ATA GCT CCG GG-3′
L200A	5′-GG AGC TAT GTG CCT GCA GCT CAT TCC AGC TC-3′<br>5′-GA GCT GGA ATG AGC TGC AGG CAC ATA GCT CC-3′
A201G	5′-GC TAT GTG CCT CTT GGC CAT TCC AGC TCA GC-3′<br>5′-GC TGA GCT GGA ATG GCC AAG AGG CAC ATA GC-3′
H202A	5′-GC TAT GTG CCT CTT GCT GCT AGC AGC TCA GCC TTC-3′<br>5′-GAA GGC TGA GCT GCT AGC AGC AAG AGG CAC ATA GC-3′
S203A	5′-G CCT CTT GCT CAT GCA TCC TCA GCC TTC ACC-3′<br>5′-GGT GAA GGC TGA GGA TGC ATG AGC AAG AGG C-3′
S204A	5′-GC TAT GTG CCT CTT GCG CAT TCC GCC TCA GCC TTC ACC-3′<br>5′-GGT GAA GGC TGA GGC GGA ATG CGC AAG AGG CAC ATA GC-3′
S205A	5′-CTT GCT CAT TCC AGC GCT GCC TTC ACC ATT AC-3′<br>5′-GT AAT GGT GAA GGC AGC GCT GGA ATG AGC AAG-3′
A206G	5′-GCT CAT TCC AGC TCC GGA TTC ACC ATT ACT GAC C-3′<br>5′-G GTC AGT AAT GGT GAA TCC GGA GCT GGA ATG AGC-3′
F207A	5′-CAT TCC AGC TCA GCT GCC ACC ATT ACT GAC C-3′<br>5′-G GTC AGT AAT GGT GGC AGC TGA GCT GGA ATG-3′
T208A	5′-CC AGC TCA GCC TTC GCG ATT ACT GAC CAG G-3′<br>5′-C CTG GTC AGT AAT CGC GAA GGC TGA GCT GG-3′
I209A	5′-GC TCA GCC TTC ACG GCT ACT GAC CAG GTG C-3′<br>5′-G CAC CTG GTC AGT AGC CGT GAA GGC TGA GC-3'
T210A	5′-GC TCA GCC TTC ACA ATT GCT GAC CAG GTG CC-3′<br>5′-GG CAC CTG GTC AGC AAT TGT GAA GGC TGA GC-3′
D211A	5′-CC TTC ACC ATT ACT GCG CAG GTG CCT TTC TCC-3′<br>5′-GGA GAA AGG CAC CTG CGC AGT AAT GGT GAA GG-3′
Q212A	5′-C ACC ATT ACT GAC GCA GTG CCT TTC TCC G-3′<br>5′-C GGA GAA AGG CAC TGC GTC AGT AAT GGT G-3′
V213A	5′-CC ATT ACT GAC CAG GCA CCT TTC TCG GTG AGC GTG TCC C-3′<br>5′-G GGA CAC GCT CAC CGA GAA AGG TGC CTG GTC AGT AAT GG-3′
P214A	5′-CC ATT ACT GAC CAG GTC GCT TTC TCC GTG AGC-3′<br>5′-GCT CAC GGA GAA AGC GAC CTG GTC AGT AAT GG-3′
F215A	5′-CT GAC CAG GTG CCT GCT AGC GTG AGC GTG TCC-3′<br>5′-GGA CAC GCT CAC GCT AGC AGG CAC CTG GTC AG-3′
S216A	5′-C CAG GTG CCT TTC GCA GTG AGC GTG TCC-3′<br>5′-GGA CAC GCT CAC TGC GAA AGG CAC CTG G-3′
V217A	5′-G GTG CCT TTC TCC GCT AGC GTG TCC CAG TTG C-3′<br>5′-G CAA CTG GGA CAC GCT AGC GGA GAA AGG CAC C-3′
S218A	5′-G CCT TTC TCC GTG GCA GTG TCC CAG TTG C-3′<br>5′-G CAA CTG GGA CAC TGC CAC GGA GAA AGG C-3′
V219A	5′-CCT TTC TCC GTG AGC GCT TCC CAG TTG CGG G-3′<br>5′-C CCG CAA CTG GGA AGC GCT CAC GGA GAA AGG-3′
S220A	5′-CCT TTC TCC GTG AGC GTT GCG CAG TTG CGG GCC TTG G-3′<br>5′-C CAA GGC CCG CAA CTG CGC AAC GCT CAC GGA GAA AGG-3′
Q221A	5′-CC GTG AGC GTG TCA GCG CTG CGG GCC TTG GAT GG-3′<br>5′-CC ATC CAA GGC CCG CAG CGC TGA CAC GCT CAC GG-3′
L222A	5′-C GTG AGC GTG TCC CAG GCG CGC GCC TTG GAT GGA GG-3′<br>5′-CC TCC ATC CAA GGC GCG CGC CTG GGA CAC GCT CAC G-3′
R223A	5′-C GTG AGC GTG TCC CAA TTG GCG GCC TTG GAT GGA GG-3′<br>5′-CC TCC ATC CAA GGC CGC CAA TTG GGA CAC GCT CAC G-3′
F149L	5′-GG TCT CAG AAG AGA AGC CTT GTT TAT GTC TGG-3′<br>5′-CCA GAC ATA AAC AAG GCT TCT CTT CTG AGA CC-3′
Y151L	5′-GG TCT CAG AAG AGA AGT TTT GTT CTT GTC TGG AAG ACC-3′<br>5′-GGT CTT CCA GAC AAG AAC AAA ACT TCT CTT CTG AGA CC-3′
Y189L	5′-CC ATG GAA GTG ACA GTA CTC CAT CGC CGG GGA TCC-3′<br>5′-GGA TCC CCG GCG ATG GAG TAC TGT CAC TTC CAT GG-3′
F207L	5′-GCT CAT TCC AGC TCA GCG CTC ACC ATT ACT GAC C-3′<br>5′-G GTC AGT AAT GGT GAG CGC TGA GCT GGA ATG AGC-3′
F215L	5′-CT GAC CAG GTG CCC TTA AGC GTG AGC GTG TCC-3′<br>5′-GGA CAC GCT CAC GCT TAA GGG CAC CTG GTC AG-3′
Y151F	5′-CT CAG AAG AGA AGC TTT GTC TTC GTC TGG AAG ACC-3′<br>5′-GGT CTT CCA GAC GAA GAC AAA GCT TCT CTT CTG AG-3′
Y189S	5′-G GAA GTG ACT GTC TCC CAT CGC CGG GGT TCC CGG AGC TAT G-3′<br>5′-C ATA GCT CCG GGA ACC CCG GCG ATG GGA GAC AGT CAC TTC C-3′
Y189F	5′-G GAA GTG ACT GTC TTC CAT CGC CGG GGT TCC CGG AGC TAT G-3′<br>5′-C ATA GCT CCG GGA ACC CCG GCG ATG GAA GAC AGT CAC TTC C-3′
$PMEL_{CAF-N}$	5′-CCT TGC CCA TCT AAG AGA TGT TGC CCG GGT TGT TGC TTT GTT TAT GTC TGG-3′<br>5′-CCA GAC ATA AAC AAA GCA ACA ACC CGG GCA ACA TCT CTT AGA TGG GCA AGG-3′
$PMEL_{CAF-C}$	5′-GC GTG TCC CAG TTG TGT TGC CCG GGT TGT TGC AAC AAG CAC TTC CTG-3′<br>5′-CAG GAA GTG CTT GTT GCA ACA ACC CGG GCA ACA CAA CTG GGA CAC GC-3′

SUPPLEMENTARY FIGURE LEGENDS

Suppl. Figure S1. Epitope-mapping of PMEL NTF-specific antibodies. The PMEL N-terminus (NTF) is excluded from melanosomal amyloid (Leonhardt et al, 2013) but was previously thought to span at least 200 amino acids. Our findings in Fig. 2A/D, however, show that it can maximally extend until Arg-147, because Ser-148 is already inside the CAF. Hence, all NTF-specific antibodies should recognize epitopes within the first 147 residues. Three such antibodies have been described (PMEL-N (Watt et al, 2009), 7E3 (Leonhardt et al, 2013), and EP4863(2) (Leonhardt et al, 2013)), but except for PMEL-N (raised against aa 24-40) (Nichols et al, 2003), their cognate epitopes are not well characterized. We used a series of sequential PMEL deletion mutants (Suppl. Fig. S1A) (Leonhardt et al, 2013) or dot blots with recombinant PMEL fragments (Suppl. Fig. S1F) to map all respective epitopes and also included one more antibody, E-7, which also reacts with the NTF (Suppl. Fig. S1B/C). Consistent with our mass spectrometry results all antibodies recognized epitopes located within the first 147 PMEL residues (Suppl. Fig. S1D-F). Specifically, we mapped the epitopes of PMEL NTF-specific antibodies to aa 28-50 (E-7), aa 28-75 (EP4863(2)), and aa 51-147 (7E3). (A) PMEL N-terminal deletion mutants (*NTF in yellow*, *CAF in blue*). (B) IF analysis using antibodies HMB50 and E-7. E-7 detects newly synthesized, early protein, which does not overlap with HMB50 labeling (mature fibrils), suggesting the NTF is not inside fibrils. (C) Western blot analysis of a lysate derived from PMEL-expressing Mel220 cells. Antibody E-7 recognizes PMEL fragments that contain the NTF. Horizontal dashed lines separate different exposures of the same blot. (D, E) IF analysis using antibodies HMB50, E-7, 7E3, and EP4863(2). The latter three antibodies recognize the NTF. Note that constructs NTR75, NTR98, NTR166, and NTR199 misfold, causing ER retention (Leonhardt et al, *Mol Biol Cell*(2013)). This likely impairs detection with conformation-sensitive antibody 7E3 (E), but does not affect E-7 or EP4863(2) labeling (D, E). Antibody HMB50 recognizes (almost) only mature fibrils in the context of wt-PMEL, but detects newly-

synthesized, non-fibrillar PMEL variants that are non-functional (see Leonhardt et al, *Mol Biol Cell*(2013) for discussion). (F) Dot blot analysis of recombinant MBP-NTF and MBP-CAF fragments using antibody 7E3.

Suppl. Figure S2. PMEL alanine-scanning mutants (newly synthesized protein) (A-J) Western blot analysis of SDS-lysed total membranes derived from Mel220 cells stably expressing PMEL alanine-scanning mutants. PMEL-specific antibodies Pep13h (A-B, D-J) and ab52058 (C), both recognizing the PMEL C-terminus, were used. Note that formation of Mβ indicates successful ER export.

Suppl. Figure S3. PMEL alanine-scanning mutants (fibrils) (A-T) Western blot analysis of SDS-lysed total membranes derived from Mel220 cells stably expressing PMEL alanine-scanning mutants. PMEL-specific antibodies HMB45 (A-K) and I51 (L-T), recognizing O-glycosylated RPT domain-containing fragments (Mα, MαC, and RPT) or the CAF, respectively, were used. Note that formation of Mα indicates successful ER export.

Suppl. Figure S4. Melanosome-lysosome segregation of PMEL CAF category 1 mutants. IF analysis of Mel220 cells stably expressing PMEL alanine-scanning mutants that have no or almost no phenotype (category 1, green in Fig. 3A). The PMEL-specific antibody HMB50 (recognizing mature fibrils) and the LAMP1-specific antibody H4A3 (recognizing a lysosomal marker) were used. In all cases, proper separation of mostly perinuclear lysosomes and largely peripheral melanosomes is observed.

Suppl. Figure S5. Subcellular distribution of PMEL CAF category 1 mutants. IF analysis of Mel220 cells stably expressing PMEL alanine-scanning mutants that have no or almost no phenotype (category 1, green in Fig. 3A). The PMEL-specific antibody HMB45 (recognizing

mature fibrils) and the PMEL-specific antibody Pep13h (recognizing newly synthesized PMEL) were used. In all cases, proper separation of newly synthesized PMEL (mostly in ER, Golgi, and stage I melanosomes) and mature PMEL (in stage II melanosomes) is observed.

Suppl. Figure S6. Fibril formation by PMEL CAF category 1 and 2 mutants. EM analysis of Epon-embedded stable Mel220 transfectants expressing PMEL alanine-scanning mutants that form fibrils (category 1, green and category 2, orange in Fig. 3A). The respective quantification of fibril formation in the indicated cell lines is shown in Suppl. Fig. 7 and summarized in Fig. 3A.

Suppl. Figure S7. Quantification of fibril formation by PMEL CAF category 1 and 2 mutants. Quantification of the EM analysis of Epon-embedded stable Mel220 transfectants expressing PMEL alanine-scanning mutants. Depicted is the number of fibril-containing organelles per cell [N=15]. A summary of these results is shown in Fig. 3A.

Suppl. Figure S8. Immunofluorescence analysis of PMEL CAF category 2 and 3 mutants. IF analysis of Mel220 cells stably expressing PMEL alanine-scanning mutants that show significantly reduced (cutoff > 55% reduction) (A, C) or no (B, D) fibril formation (category 2, orange and category 3, red in Fig. 3A). (A, B) The PMEL-specific antibody HMB45 (recognizing mature fibrils) and the PMEL-specific antibody Pep13h (recognizing newly synthesized PMEL) were used. Note that in some of the category 2 cases and in all of the category 3 cases the separation of the two labelings is incomplete, suggesting that fibril formation is impaired or abrogated (Leonhardt et al, *Mol Biol Cell*(2013)). Exceptions are F149A and I172A, which do not react with HMB45, because they are not exported from the ER (F149A) (HMB45 recognizes an O-glycosylated epitope) or because Mα likely undergoes early degradation (I172A). (C, D) The PMEL-specific antibody HMB50 (recognizing mature fibrils) and the LAMP1-specific antibody H4A3 (recognizing a lysosomal marker) were used. Note that in some but not all of the category

2 cases (as well as for Y189A) there is extensive co-localization of the two labelings in the perinuclear region, a phenotype that we previously associated with impaired fibril formation or with the formation of non-fibrillar aggregates (Leonhardt et al, *Mol Biol Cell*(2013); Leonhardt et al, *J Biol Chem*(2010)).

Suppl. Figure S9. Immunofluorescence and electron microscopy analysis of PMEL leucine and phenylalanine substitution mutants. (A) IF analysis of Mel220 cells stably expressing PMEL leucine (*columns 1-3 and 7-9*) or phenylalanine (*columns 4-6 and 10-12*) substitution mutants. Either a combination of the PMEL-specific antibody HMB45 (recognizing mature fibrils) and the PMEL-specific antibody Pep13h (recognizing newly synthesized PMEL) (*columns 1-6*) or a combination of the PMEL-specific antibody HMB50 (recognizing mature fibrils) and the LAMP1-specific antibody H4A3 (recognizing a lysosomal marker) (*columns 7-12*) were used. (B, C, D) EM analysis of Epon-embedded stable Mel220 transfectants expressing PMEL leucine (B) or phenylalanine (C, D) substitution mutants. The respective quantification of fibril formation in the indicated cell lines is shown in Fig. 4D/E/I/J.

Suppl. Figure S10. Co-expression of functional (D211A) with loss-of-function PMEL mutants and prediction of amyloidogenic segments in the PMEL CAF. (A) Confirmation of co-expression of PMEL constructs in the cell lines analyzed in Fig. 5. cDNA was prepared from all indicated Mel220 transfectants co-expressing functional PMEL-D211A together with a PMEL loss-of-function mutant. Using this cDNA as a template, a PCR fragment encompassing the DNA region that encodes the respective loss-of-function mutation was synthesized and sequenced. As a control, a corresponding PCR fragment was synthesized using cDNA isolated from cells expressing PMEL-D211A alone. Note that co-expression of PMEL-D211A with loss-of-function mutants is reflected in sequence histograms by the overlap of two peaks in positions that are divergent between the two PMEL constructs. The expected DNA sequences for PMEL-D211A

and for the respective loss-of-function mutants are depicted for each histogram. (B) CAF sequences from 19 species were subjected to the six indicated amyloid prediction algorithms. Consensus sequences are given at the bottom of each alignment (X indicates positions predicted to be part of an amyloidogenic segment in at least 15 of the 19 species). Segments with predicted amyloidogenicity are highlighted in red.

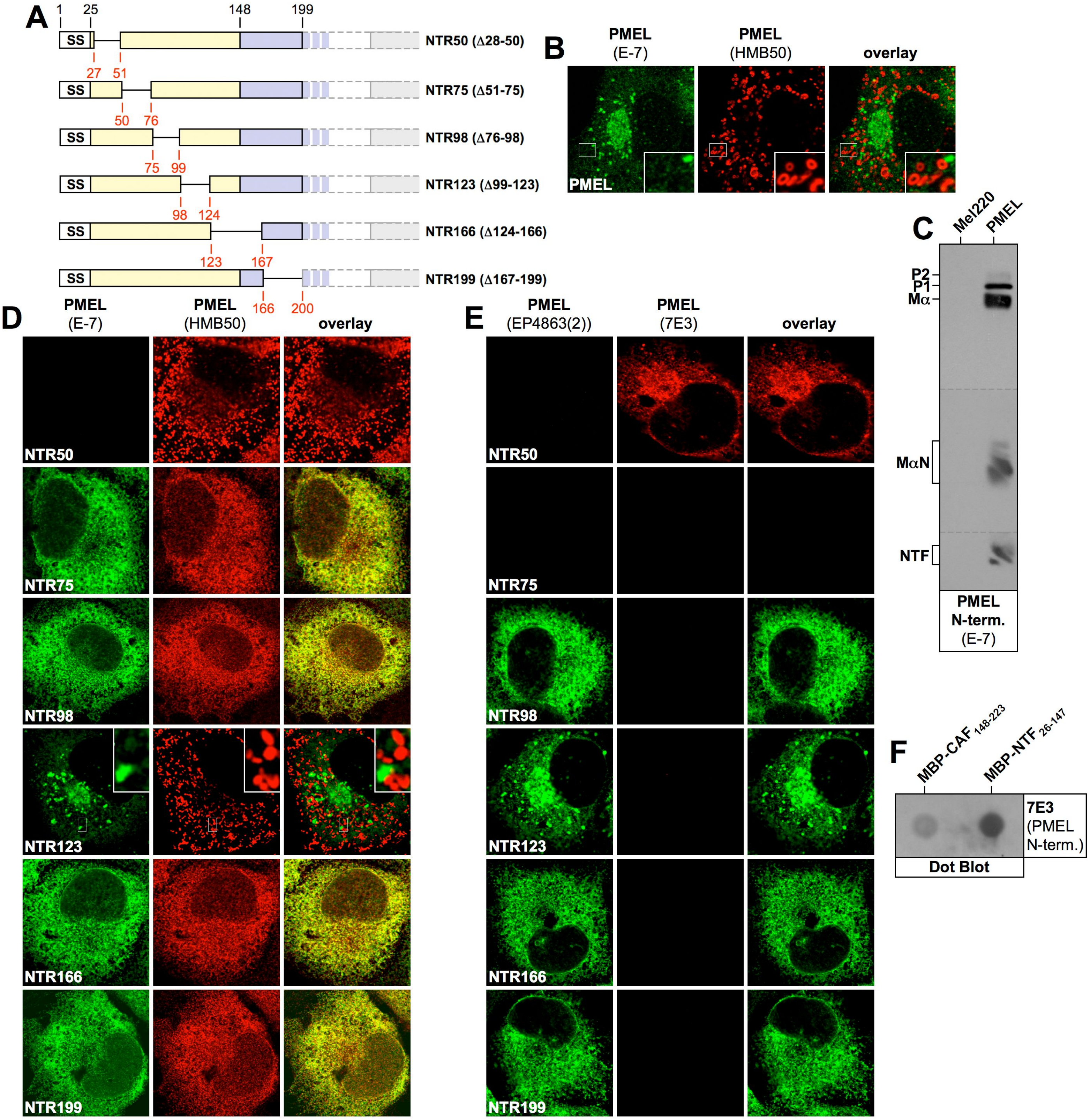

Supplemental Figure S1

Supplemental Figure S2

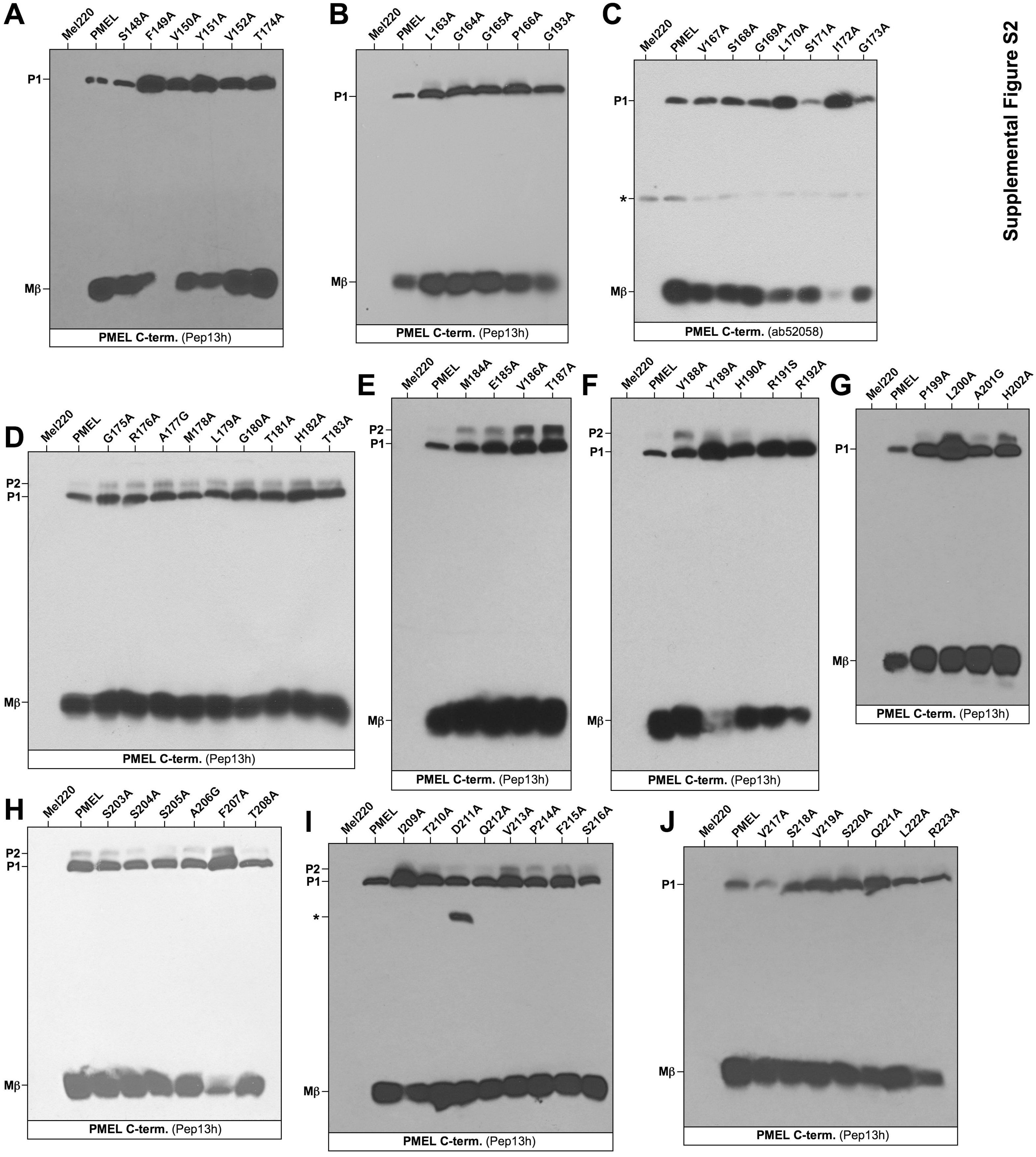

Supplemental Figure S3

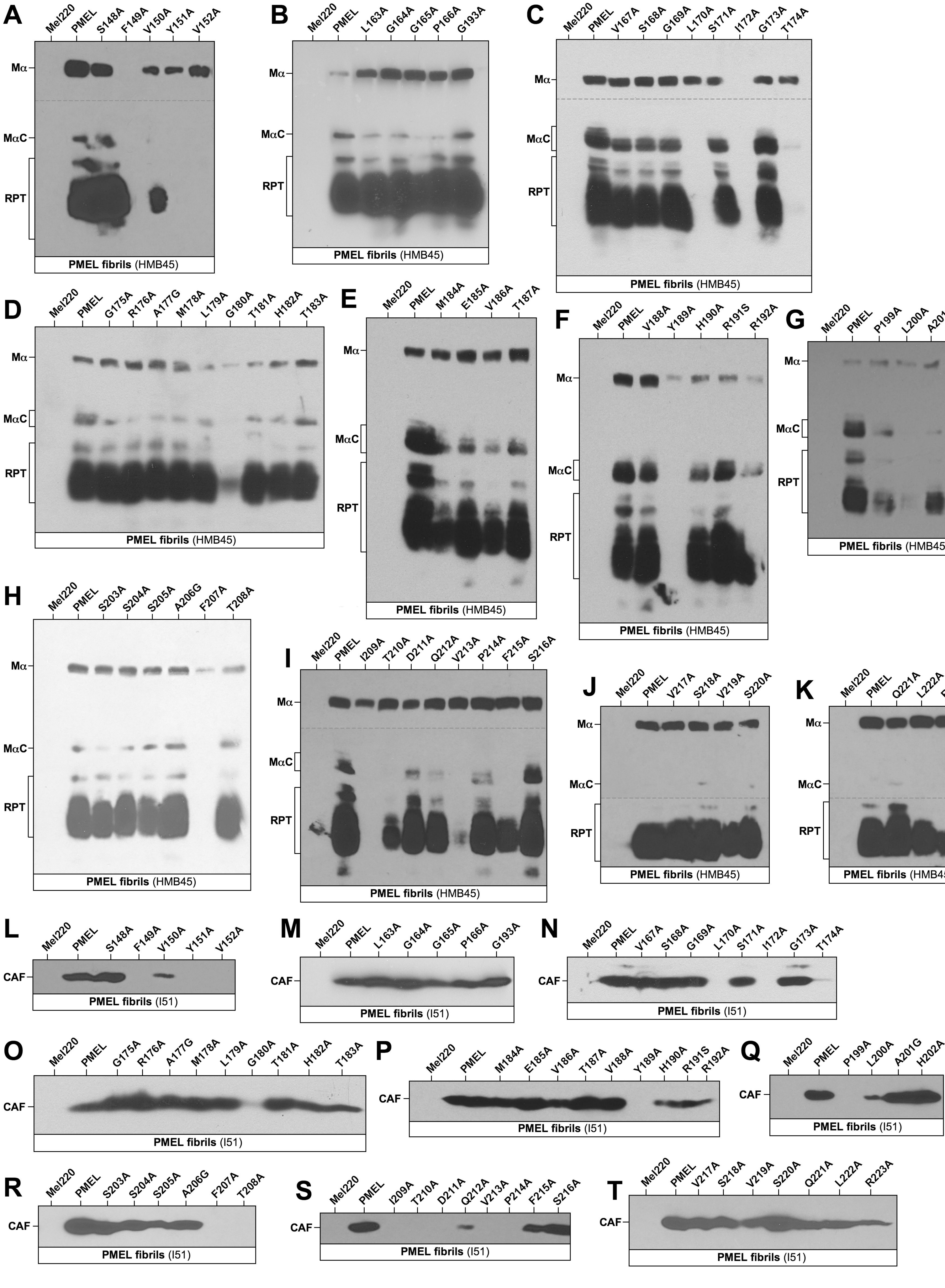

Supplemental Figure S4

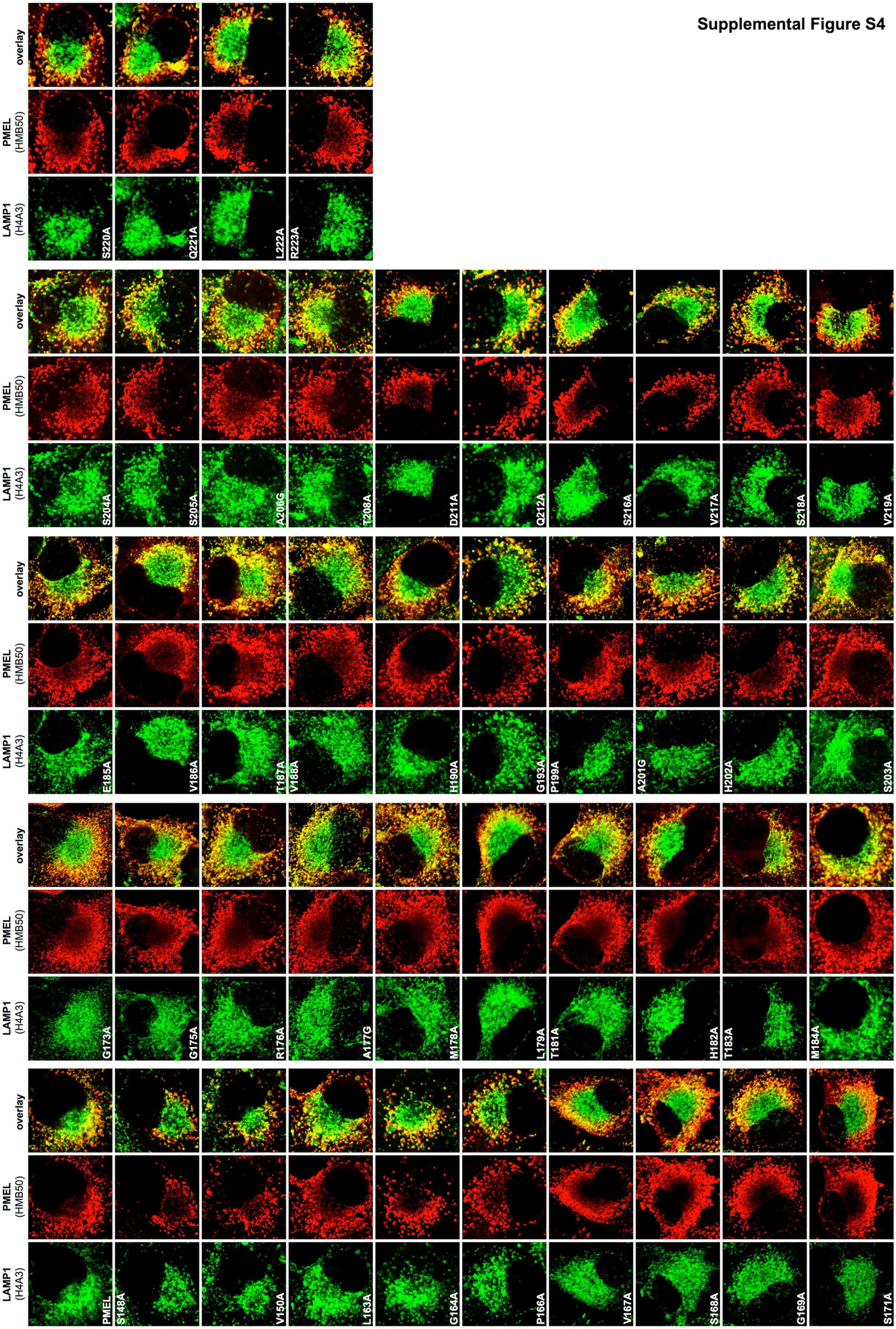

Supplemental Figure S5

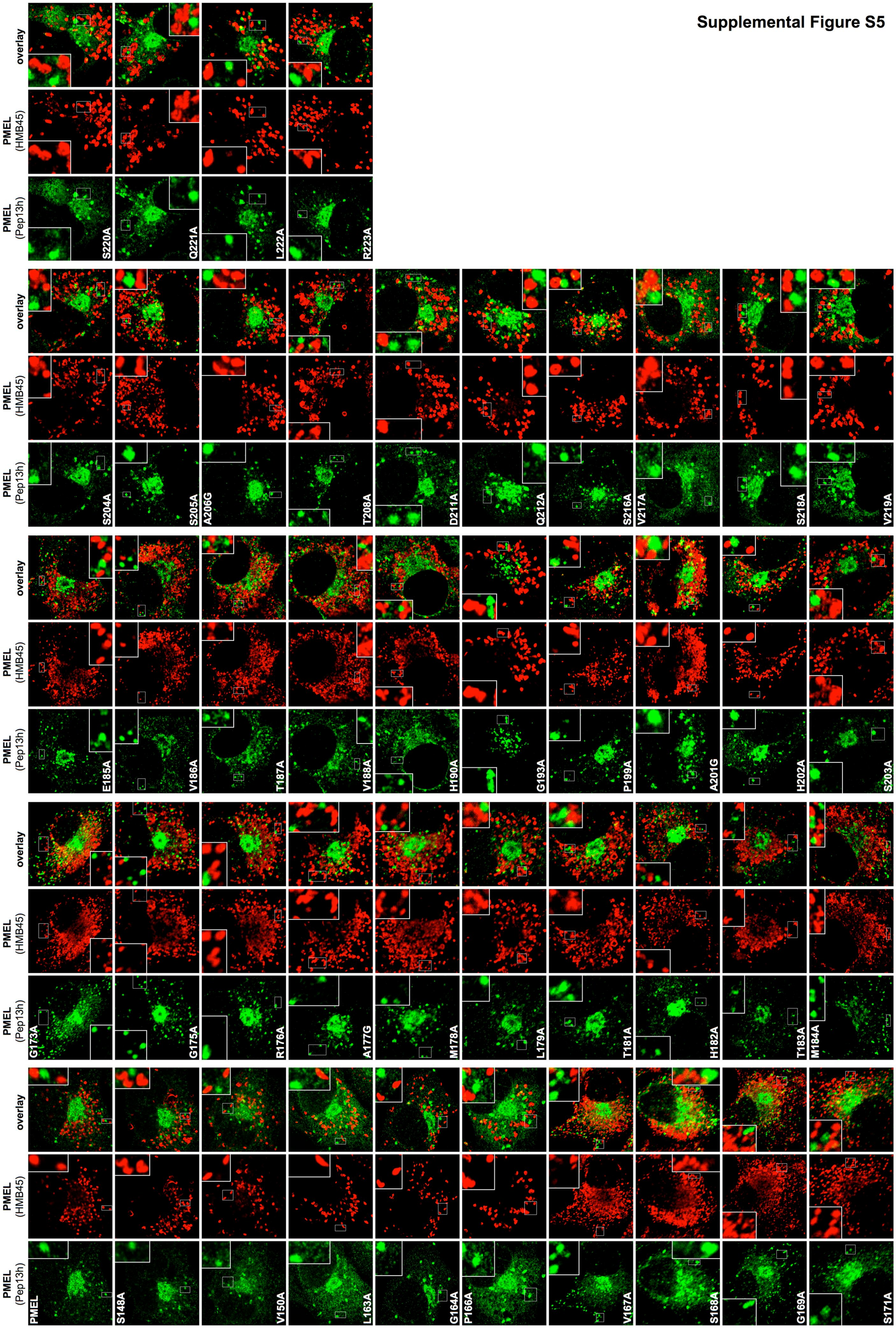

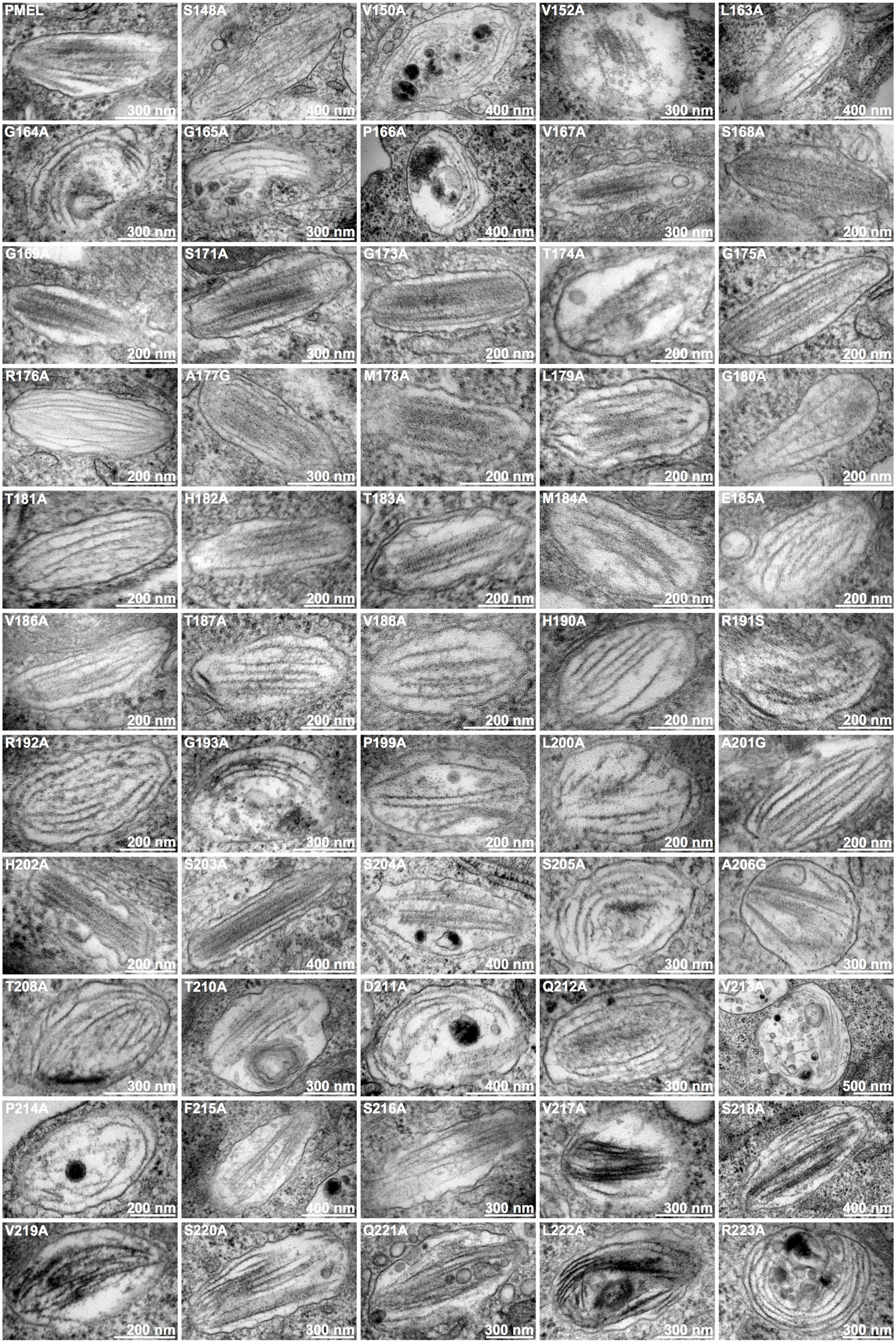

Supplemental Figure S6

Supplemental Figure S7

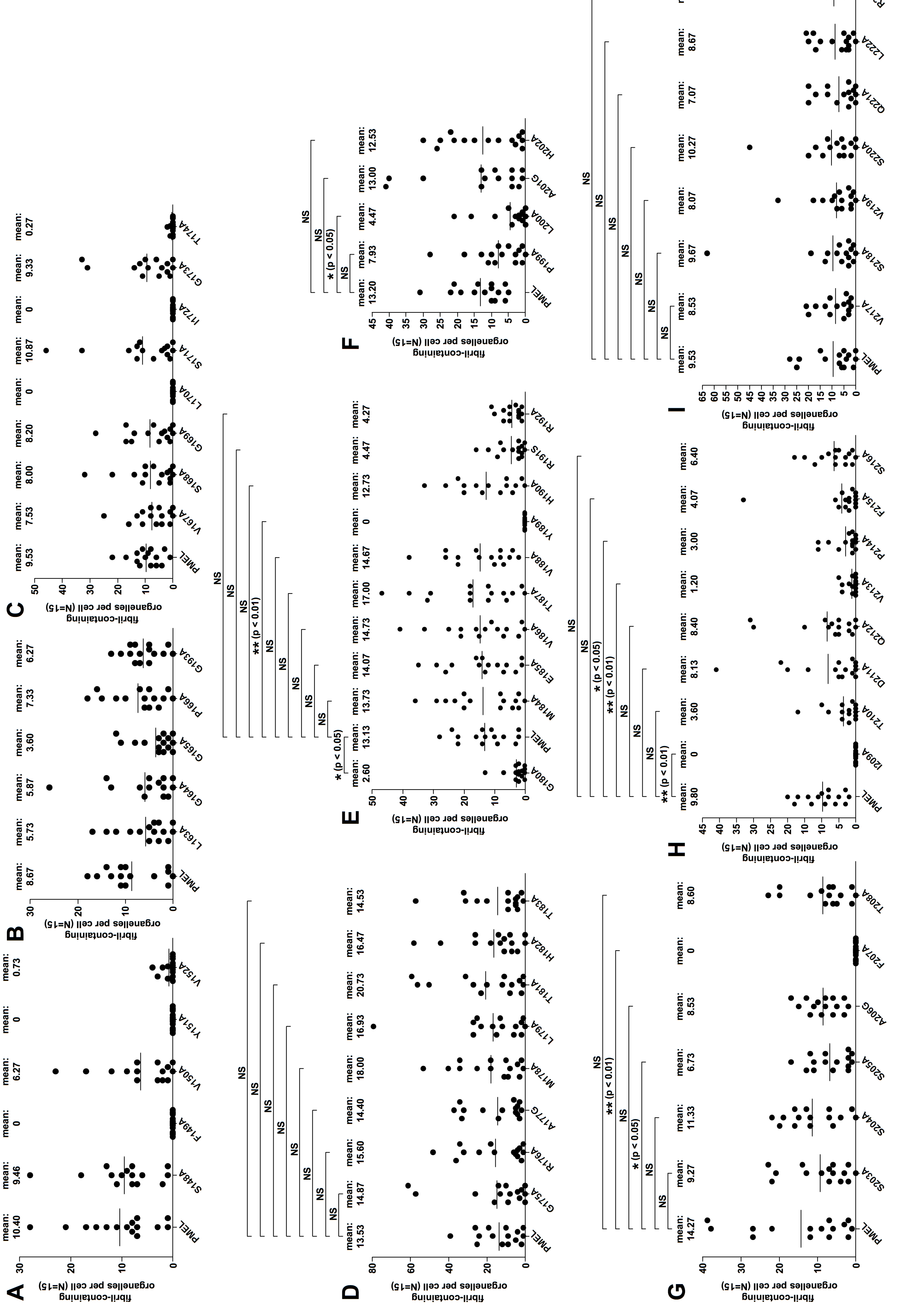

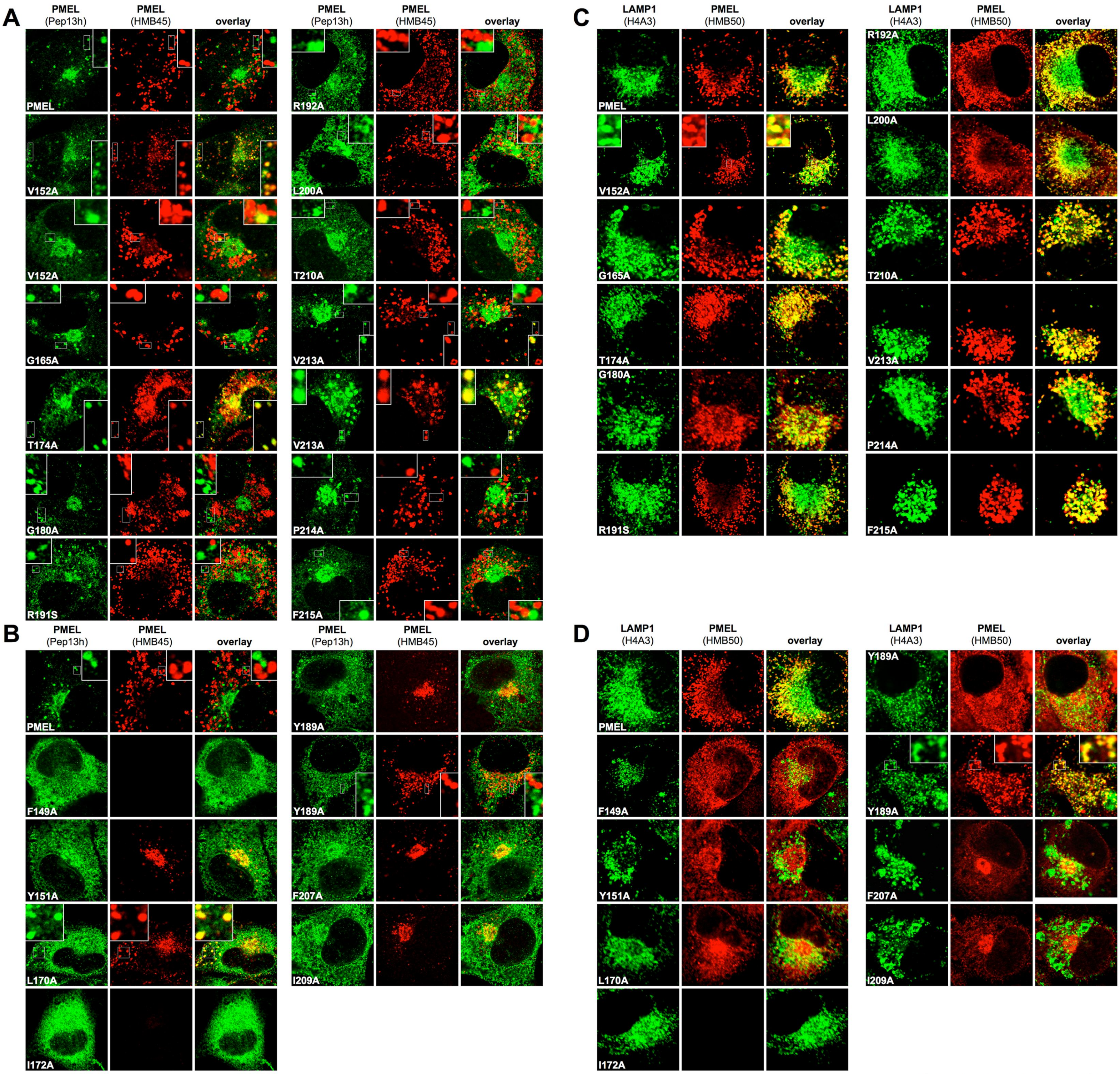

Supplemental Figure S8

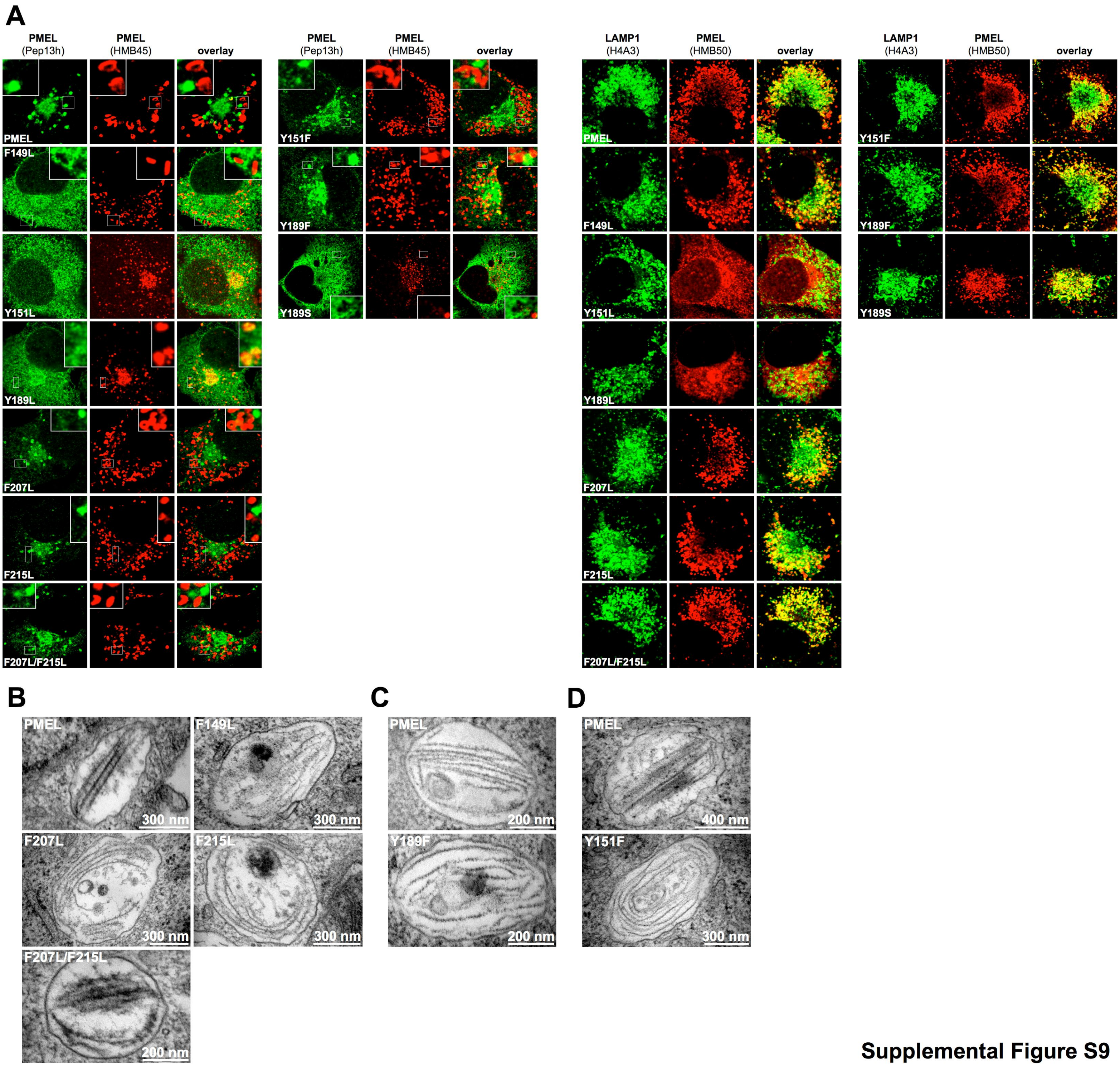

Supplemental Figure S9

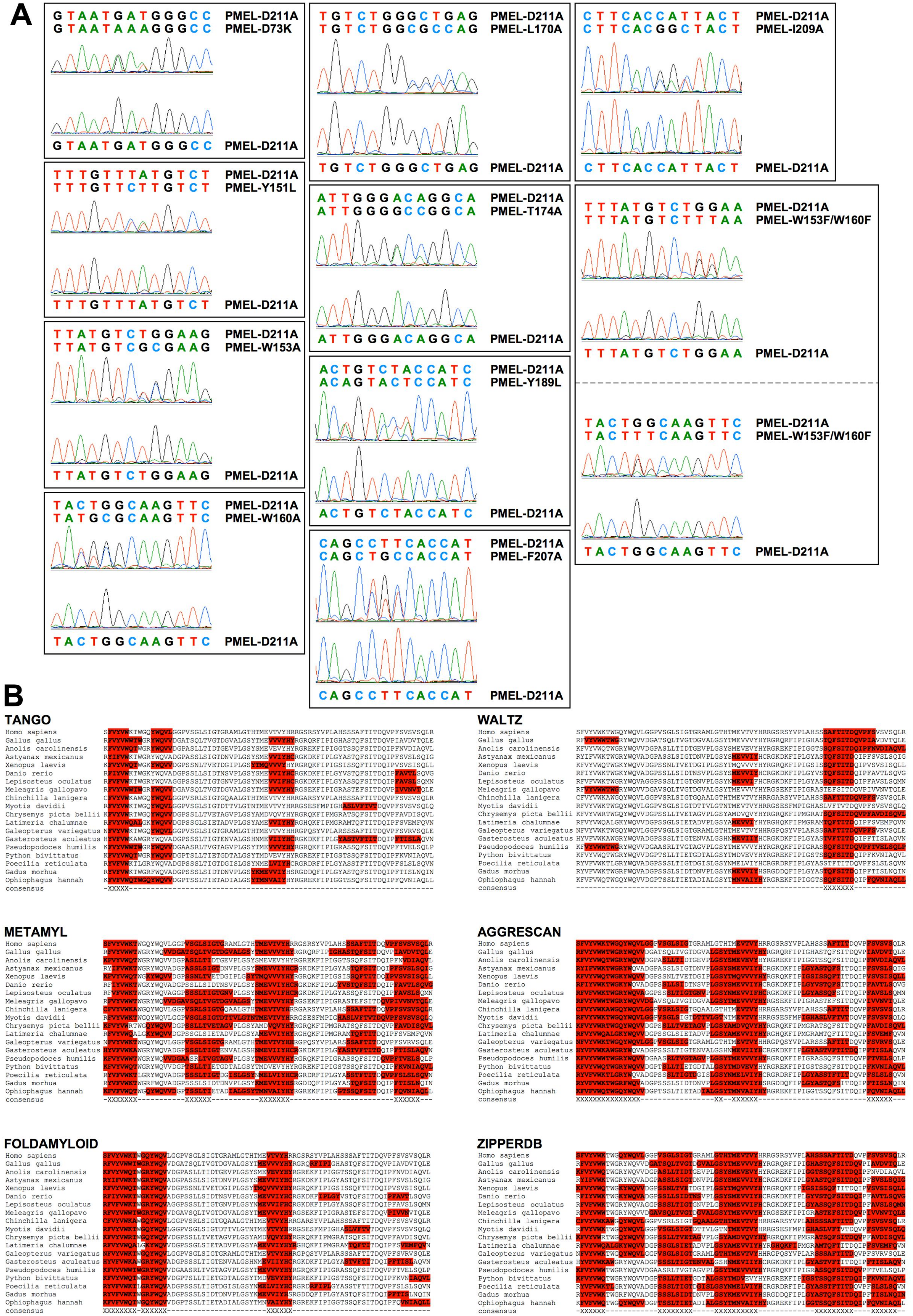

Supplemental Figure S10
